# Survival of gastric cancer in China from 2000 to 2022: A nationwide systematic review of hospital-based studies

**DOI:** 10.7189/jogh.12.11014

**Published:** 2022-12-17

**Authors:** Houqiang Li, Han Zhang, Hujia Zhang, Youxin Wang, Xiaobing Wang, Haifeng Hou, Global Health Epidemiology Reference Group

**Affiliations:** 1 School of Public Health, Shandong First Medical University & Shandong Academy of Medical Sciences, Jinan, China; 2 Beijing Key Laboratory of Clinical Epidemiology, School of Public Health, Capital Medical University; 3 Cancer Hospital, Chinese Academy of Medical Sciences; 4 School of Public Health and The Second Affiliated Hospital of Shandong First Medical University, Taian, China

## Abstract

**Background:**

Gastric cancer (GC) mortality continues to fall in industrialized countries, but still remains a public health concern in China, accounting for more than 370 000 deaths. We aimed to evaluate the survival of GC in China from 2000 to 2022 through a nationwide systematic review of hospital-based studies and to identify whether hospital-based studies show higher survival rates than population-based studies.

**Methods:**

We searched PubMed, Embase, Web of Science, and the Chinese databases of CNKI and Wanfang for hospital-based studies on GC survival published between January 1, 2000, and January 20, 2022. We calculated the nationwide GC survival rate (SR) and its 95% confidence interval (CI) and conducted subgroup analyses on histologic type, subsite, tumour node metastasis (TNM) stage, therapy type, study design, and participant region. The study protocol was registered in PROSPERO (CRD-42019121559).

**Results:**

The initial literature search returned 36 613 publications, among which 664 studies (180 798 participants) matched the inclusion criteria and were included in the meta-analysis. The pooled one-, two-, three- and five-year SRs of GC were 75.4% (95% CI = 74.0%-76.8%), 54.3% (95% CI = 50.1%-58.6%), 53.4% (95% CI = 50.4%-56.4%), and 44.5% (95% CI = 41.5%-47.5%), respectively. Subgroup analyses revealed an increase in three- and five-year SRs from 2006 to 2022. The five-year SR was highest among patients without lymph node metastasis (pooled SR = 67.8%, 95% CI = 62.8%-72.7%) and lowest among those with distant metastasis (pooled SR = 8.4%, 95% CI = 5.1%-11.7%).

**Conclusions:**

Our findings illustrate that the long-term survival of GC has improved in China since 2000. Hospital-based studies have presented higher SRs than population-based surveillance.

Gastric cancer (GC) is one of the most common public health burdens globally, particularly in east Asia (China, Japan, Korea, and Mongolia). According to Global Cancer Statistics 2020, more than 1 089 103 new GC cases and 768 793 GC-induced deaths were recorded, ranking fifth in incidence and fourth in mortality among all cancer types [1]. A continuous downward trend of incidence and mortality has been observed in industrialized countries [2-4]. Meanwhile, the death toll from GC remains high in China due to accelerated population ageing [5]. The main cause of GC, *Helicobacter pylori* infection, remains fairly common [6]. Other modifiable determinants (eg, cigarette smoking, alcohol consumption, excess body weight, diet, and health care access) will have enduring effects on the GC epidemic [7]. The classification systems by the World Health Organization (WHO) and Laurén have been widely used to distinguish histopathological types of GC. Under the WHO’s standards, gastric adenocarcinoma (GA) can be classified into subtypes including tubular, papillary, mucinous, signet ring cell, and others [8], which are of higher complexity [5].

In China, GC ranks as the third leading death burden from malignancies behind lung and liver cancers [1,3,7]. The condition accounted for 373 789 deaths in 2020 [9]. Additionally, GC causes the second highest disability-adjusted life year burden (9 824 993 years), comprising 14.6% of all cancers [10]. GC mortality decreased by 3.8% in rural areas and 2.3% in urban areas of China between 2003 and 2015, implying a turning point [11]. Yet GC incidence is much higher in China than in the United States and Europe [10]. In some east Asian countries (eg, Japan and Korea), early screening and targeted treatment have been conducive to reducing GC-related mortality and public health burdens [12-14]. The National Upper Gastrointestinal Cancer Early Detection programme, composed of screening programmes for GC and oesophageal cancer, achieved remarkable success in GC prevention in China [15]. The country’s cancer surveillance system (a population-based national cancer registry launched in 2002) covered 598 million persons in 1152 counties or districts in China as of 2020 [16]. Despite obvious priorities in collecting comprehensive cancer data [17], population-based cancer registries easily yield bias in survival detection due to incomplete follow-up [18]. By contrast, hospital-based cancer registries provide more details about clinical features, treatment approaches, and long-term prognosis, thereby contributing to well-designed survival analysis [19]. We conducted a comprehensive pooled analysis of hospital-based studies to evaluate GC survival in China and aimed to identify whether hospital-based studies show higher survival rates than population-based studies.

## METHODS

We conducted this systematic review and meta-analysis following PRISMA guidelines (Table S1 in the **Online Supplementary Document**). We registered the study protocol was registered in PROSPERO: CRD42022306143.

### Data sources and search strategy

A systematic literature search was carried out in two common Chinese research databases (CNKI and Wanfang) and three international databases (PubMed, Embase, and Web of Science). We employed several academic terms (“gastric cancer,” “gastric carcinoma,” “stomach neoplasms,” “China,” “Chinese,” “hospital,” “survival”) to search for original studies on GC survival published between January 1, 2000, and January 20, 2022. We also screened the citations of relevant articles to identify additional studies. The full search strategy is detailed in **Table S2 in the** **Online Supplementary Document**.

### Eligibility criteria and quality assessment

Two reviewers (HZ and HJZ) independently screened titles and abstracts for eligibility, after which they checked the full text and supplementary data of all retrieved publications. All disagreements were resolved by discussion between the two authors and/or through arbitration with the third professional investigator (HL). Quality appraisal was performed using a methodology quality scale (Table S3 in the **Online Supplementary Document**) designed in accordance with Newcastle-Ottawa Scale (NOS), by which studies with a score ≥4 were included [20].

The inclusion criteria for the retrieved studies were as follows: 1) GC was diagnosed based on pathology reports, 2) data were from hospital-based studies, 3) survival data of GC patients were available, 4) study participants were ethnically Chinese, and 5) the studies adopted a retrospective and prospective cohort design. Exclusion criteria were as follows: 1) in vitro experiments or animal studies, 2) reviews, comments, conference abstracts, or case reports, 3) studies of population-based databases, 4) community-based surveys, 5) survival rates (SRs) of GC or number of survived cases not available, 6) results of survival data not available, and 7) low-quality studies (score <4).

### Data extraction

The following features of selected sources were collected: 1) publication information (ie, first author’s name, year and location of included studies), 2) study design (ie, retrospective or prospective), 3) characteristics of the study population (ie, region of hospital, tumour location and specific area of stomach, tumour node metastasis (TNM) stage, clinical type, therapy, Borrmann classification, and tumour metastasis), and 4) SRs at one, two, three, and five years or the number of patients alive during follow-up. The largest and most recently published studies were included only when several studies involved the same participants.

### Statistical analysis

We performed all statistical analyses using Stata/MP 16.0 software (Stata Corp CLL, College Station, TX 77845, USA). We applied Cochran’s Q test and the *I^2^* statistic to estimate heterogeneity across studies. Assuming that heterogeneity is not significant (*P* > 0.10 or *I*^2^<50%), we used the fixed-effects model to calculate pooled SRs and their 95% confidence intervals (CIs). Otherwise, heterogeneity was statistically significant and the random-effects model was employed using the Der Simonian-Laird method [21]. We conducted subgroup analyses according to the population characteristics, GC features, study design, hospital area, and time frame of included studies (ie, 2000-2005, 2006-2010, 2011-2015, 2016-2022). We carried out the sensitivity analysis by removing one study at a time and evaluated potential publication bias via funnel plots and Egger’s test. Results with *P* < 0.05 were considered statistically significant.

## RESULTS

**Figure 1:**
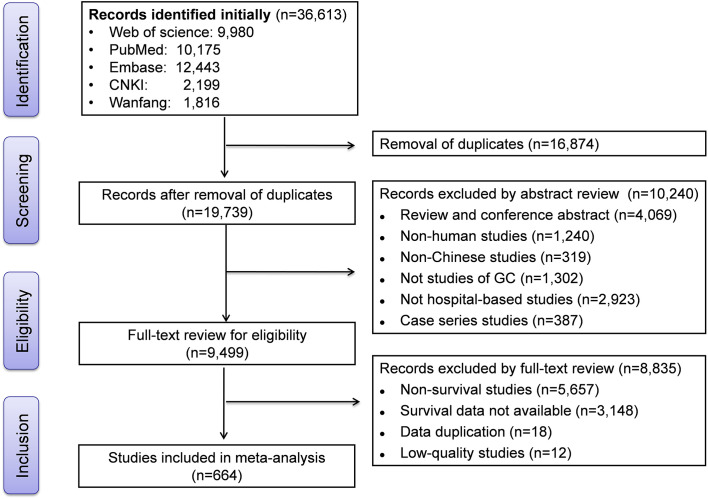
Flow diagram of literature screening.

The initial literature search retrieved 36 613 studies (10 175 in PubMed, 12 443 in Embase, 9980 in Web of Science, 2199 in CNKI, and 1816 in Wanfang). After removing 16 874 duplicates, we reviewed the title, abstract, or full text of 19 739 articles. Finally, 664 studies that met eligibility criteria were retained for the meta-analysis. The flowchart showing the literature screening process is available in **Figure 1**.

### Characteristics of included studies

**Figure 2:**
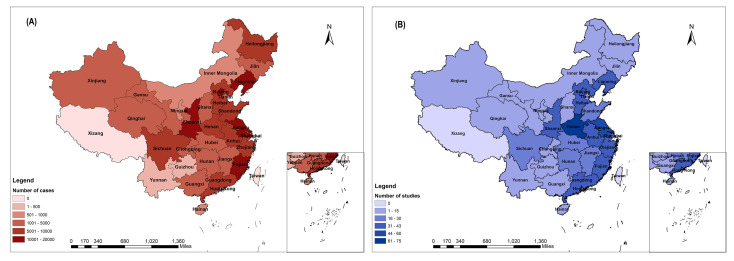
Geographical distribution of the included patients and studies. **Panel A:** distribution of patients. **Panel B:** distribution of included studies

We evaluated the survival of 180 798 participants (97 701 men, 44 609 women, 38 488 with no gender available; mean age = 58.8 years) from the 664 studies included in the meta-analysis. As shown in **Figure 2**, 188 studies investigated GC survival in high-risk regions of China (ie, Shandong, Gansu, Liaoning, Jiangsu, and Fujian) whereas 476 covered other areas. The characteristics and methodology qualities of included studies are presented in Table S4 in the **Online Supplementary Document**.

### Pooled survival of GC

Pooled SRs were 75.4% (95% CI = 74.0%-76.8%) at one year, 54.3% (95% CI = 50.1%-58.6%) at two years, 53.4% (95% CI = 50.4%-56.4%) at three years, and 44.5% (95% CI = 41.5%-47.5%) at five years (**Table 1**). We observed significant heterogeneity across the original studies for one-year (*I*^2^ = 97.4%, *P* < 0.001), two-year (*I^2^* = 98.5%, *P* < 0.001), three-year (*I*^2^ = 99.1%, *P* < 0.001), and five-year survival (*I^2^* = 99.4%, *P* < 0.001).

**Table 1:** Pooled survival rates and 95% CI of gastric cancer in high-prevalence areas

Survival	High prevalence areas		Other areas		Overall	
	**N**	**SR (%)**	**N**	**SR (%)**	**N**	**SR (%)**
One-year	96	77.8 (75.4-80.3)	273	74.6 (72.9-76.3)	369	75.4 (74.0-76.8)
Two-year	44	56.3 (48.0-64.7)	128	53.7 (48.9-58.4)	172	54.3 (50.1-58.6)
Three-year	91	52.1 (47.1-57.0)	247	53.8 (50.2-57.5)	338	53.4 (50.4-56.4)
Five-year	83	44.3 (40.1-48.5)	212	44.6 (40.7-48.4)	295	44.5 (41.5-47.5)

N – numbers of included studies, SR – survival rate, CI – confidence interval

### Subgroup analyses by study design

All included studies were classified as either prospective or retrospective. Pooled one-, two-, three-, and five-year SRs were 74.7% (95% CI = 72.4%-77.0%), 55.6% (95% CI = 49.4%-61.8%), 54.3% (95% CI = 50.0%-56.4%), and 40.7% (95% CI = 33.9%-47.6%), respectively, among 241 prospective studies. Pooled one-, two-, three-, and five-year SRs were 75.8% (95% CI = 74.0%-77.7%), 53.4% (95% CI = 47.7–59.1%), 52.9% (95% CI = 49.0%-56.8%), and 45.5% (95% CI = 42.2%-48.8%), respectively, among 423 retrospective studies (**Table 2**).

**Table 2:** Pooled survival rates (%) of gastric cancer in retrospective and prospective

Survival	Retrospective studies			Prospective studies	
	**N**	**SR (95% CI)**		**N**	**SR (95% CI)**
One-year	218	75.8 (74.0-77.7)		151	74.7 (72.4-77.0)
Two-year	97	53.4 (47.7-59.1)		75	55.6 (49.4-61.8)
Three-year	225	52.9 (49.0-56.8)		113	54.3 (50.0-56.4)
Five-year	232	45.5 (42.2-48.8)		63	40.7 (33.9-47.6)

N – numbers of included studies, SR – survival rate, CI – confidence interval

### Subgroup analyses by period

**Figure 3:**
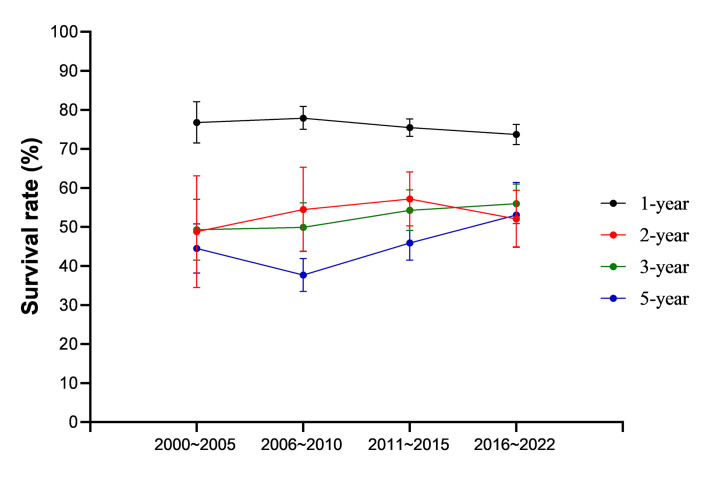
Time-trend graph of the gastric cancer overall survival rates from 2000 to 2022.

We performed a time-trend analysis to detect trends in GC survival from 2000 to 2022. Findings showed that three- and five-year SRs increased between 2006 and 2022 (**Figure 3** and Table S5 in the **Online Supplementary Document**). However, we observed a negative trend toward for survival at one-year prognosis from 2006 to 2022.

### Subgroup analyses by participant region

A total of 188 studies reported GC survival in high-risk areas (ie, Shandong, Gansu, Liaoning, Jiangsu, and Fujian): the pooled SRs at one, two, three, and five years were 77.8% (95% CI = 75.4%-80.3%), 56.3% (95% CI = 48.0%-64.7%), 52.1% (95% CI = 47.1%-57.0%), and 44.3% (95% CI = 40.1%-48.5%), respectively (**Table 1**). Pooled one- and two-year SRs were greater in high-risk areas, indicating better clinical management in these regions.

### Subgroup analyses by clinical features

**Figure 4:**
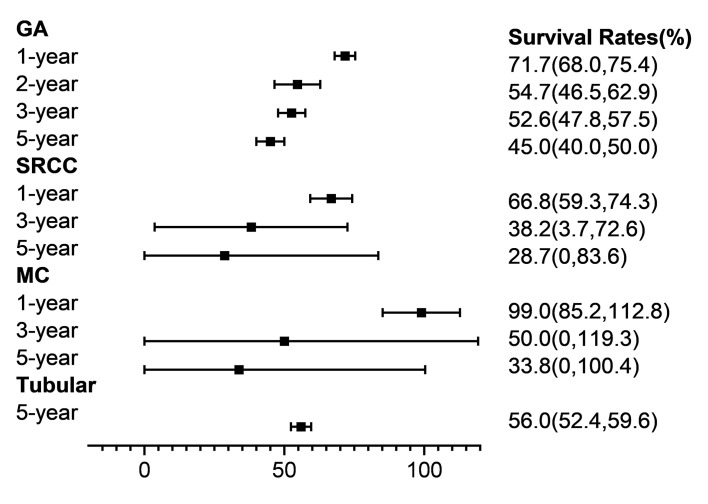
Forest plot of histological types of gastric cancer. GA – gastric adenocarcinoma, SRCC – signet ring cell carcinoma, MC – mucinous adenocarcinoma, Tubular – tubular adenocarcinoma.

We also conducted subgroup analyses by tumour type classification, TNM stage, tumour location, therapy, Borrmann classification, and metastasis status (**Table 3**, **Figure 4**). The pooled SRs of GA were 71.7% (95% CI = 68.0%-75.4%) at one year, 54.7% (95% CI = 46.5%-62.9%) at two years, 52.6% (95% CI = 47.8%-57.5%) at three years, and 45.0% (95% CI = 40.0%-50.0%) at five years. These proportions were significantly higher than those for signet ring cell carcinoma.

**Table 3:** Pooled survival rates (%) of gastric cancer in retrospective and prospective

Categories		One-year		Two-year		Three-year		Five-year	
		**N**	**SR (95% CI)**	**N**	**SR (95% CI)**	**N**	**SR (95% CI)**	**N**	**SR (95% CI)**
**Histological type**	GA	64	71.7 (68.0-75.4)	33	54.7 (46.5-62.9)	58	52.6 (47.8-57.5)	69	45.0 (40.0-50.0)
	SRCC	2	66.8 (59.3-74.3)	NA	NA	3	38.2 (3.7-72.6)	2	28.7 (0-83.6)
	MC	1	99.0 (85.2-112.8)	NA	NA	1	50.0 (0-119.3)	2	33.8 (0-100.4)
	Tubular	NA	NA	NA	NA	NA	NA	1	56.0 (52.4-59.6)
									
**TNM stage**	I	16	98.4 (97.4-99.3)	5	81.0 (67.6-94.5)	25	91.3 (88.6-94.0)	41	83.9 (81.2-86.7)
	II	19	94.2 (92.2-96.3)	8	73.2 (61.7-84.7)	28	73.2 (68.5-77.9)	45	61.5 (58.0-64.9)
	III	26	80.6 (77.0-84.3)	13	54.0 (36.5-71.5)	35	43.6 (39.8-47.4)	56	31.9 (28.9-34.8)
	IV	32	54.2 (45.9-62.5)	18	24.6 (15.7-33.5)	25	12.3 (8.1-16.5)	28	9.1 (6.5-11.7)
	I/II	4	95.9 (92.7-99.2)	1	96.0 (91.5-100.5)	6	71.0 (54.0-88.0)	13	65.7 (55.6-75.8)
	II/III	15	84.0 (80.0-88.0)	7	64.9 (56.5-73.3)	19	56.2 (50.8-61.5)	7	50.5 (30.7-70.3)
	III/IV	23	60.4 (49.8-70.9)	17	43.2 (31.7-54.6)	14	38.2 (24.7-51.6)	12	29.6 (17.8-41.4)
	I/II/III	7	93.8 (90.4-97.2)	3	83.5 (70.9-96.1)	8	66.2 (57.4-75.0)	5	45.9 (13.6-78.2)
	II/III/IV	10	74.8 (63.2-86.4)	4	70.6 (64.9-76.3)	6	51.9 (41.2-62.7)	3	46.7 (39.9-53.4)
	I/II/III/IV	2	72.5 (53.9-91.1)	2	75.3 (67.4-83.1)	3	50.2 (25.9-74.4)	3	47.7 (0.6-94.1)
									
**Borrmann style**	I	5	93.2 (87.8-98.5)	2	96.4 (90.7-102.2)	6	75.0 (60.5-89.4)	16	55.9 (43.1-68.7)
	II	5	91.8 (88.0-95.6)	2	74.6 (55.0-94.2)	6	73.4 (65.5-81.2)	16	52.3 (45.2-59.5)
	III	5	77.6 (61.4-93.8)	2	77.7 (67.9-87.5)	6	47.7 (31.9-63.4)	16	35.7 (29.1-42.3)
	IV	5	73.0 (49.6-96.4)	2	50.0 (23.8-76.2)	6	29.2 (22.8-35.5)	17	20.5 (15.0-26.0)
	I/II	NA	NA	1	62.0 (52.5-71.5)	2	87.6 (82.3-92.9)	3	59.6 (50.5-68.7)
	II/III	NA	NA	NA	NA	NA	NA	1	55.0 (49.5-60.5)
	III/IV	NA	NA	1	45.0 (37.7-52.3)	2	45.5 (38.4-52.7)	3	38.1 (25.2-51.1)
									
**Location**	Upper	14	84.6 (80.1-89.2)	6	64.5 (54.5-74.6)	24	52.7 (46.4-58.9)	40	43.6 (38.7-48.5)
	Middle	10	96.4 (80.1-92.8)	5	61.2 (41.2-81.1)	14	57.8 (51.4-64.3)	36	47.6 (42.1-53.1)
	Lower	14	88.4 (85.0-91.8)	5	68.2 (57.4-78.9)	20	61.1 (53.7-68.5)	36	51.5 (46.5-56.5)
	Entire	3	76.1 (67.0-85.2)	3	22.4 (0.3-44.5)	4	55.5 (23.0-87.9)	9	22.6 (18.6-26.5)
	Mixed	4	70.6 (48.4-92.8)	1	67.0 (54.8-79.2)	7	46.4 (27.0-65.9)	12	36.1 (24.8-47.4)
	EGJ	3	76.3 (42.6-110.1)	2	45.4 (0-97.3)	8	49.3 (32.8-65.9)	10	47.2 (22.5-71.9)
									
**Site**	Cardia	6	84.8 (76.2-93.4)	2	70.2 (40.8-99.6)	10	48.1 (37.9-58.2)	11	45.5 (35.0-56.0)
	Cardia/Fundus	1	87.0 (79.6-94.4)	1	60.0 (46.0-74.0)	2	54.9 (35.7-74.2)	3	52.7 (45.1-60.2)
	Fundus	1	97.0 (93.6-100.4)	NA	NA	2	56.8 (31.4-82.3)	3	42.9 (17.6-68.1)
	Body	7	87.4 (79.8-94.9)	3	73.1 (53.8-92.5)	10	53.3 (42.9-63.7)	17	43.9 (37.7-50.1)
	Body/Antrum	NA	NA	1	67.0 (54.8-79.2)	2	61.5 (51.7-71.3)	NA	NA
	Antrum	9	86.3 (80.5-92.1)	3	71.0 (52.5-89.5)	1	58.8 (48.3-69.3)	15	48.6 (40.7-56.6)
	Angle	NA	NA	NA	NA	1	81.0 (65.9-96.1)	1	27.0 (9.9-44.1)
**Gastric stump**									
		19	69.5 (59.8-79.2)	2	32.8 (46.3-49.4)	19	38.2 (30.6-45.9)	17	22.2 (15.6-28.8)
									
**Metastasis**	N0	13	93.4 (90.6-96.3)	1	98.0 (94.2-101.8)	24	78.6 (72.9-84.4)	45	67.8 (62.8-72.7)
	N1/N2/N3	17	74.2 (67.7-80.8)	4	43.1 (21.2-65.0)	27	49.7 (42.3-57.1)	55	39.6 (35.0-43.6)
	M1	30	53.4 (43.7-63.0)	15	24.7 (12.7-36.7)	18	16.5 (10.3-22.6)	18	8.4 (5.1-11.7)
									
**Therapy**	Surgery (S)	137	82.4 (80.6-84.2)	59	62.5 (54.9-70.0)	149	57.5 (53.3-61.6)	157	46.3 (42.9-49.8)
	Chemotherapy (C)	53	61.0 (54.9-67.0)	26	46.2 (35.7-56.7)	26	53.7 (47.5-59.8)	16	42.3 (30.9-53.6)
	Radiation (R)	2	38.9 (0-81.1)	NA	NA	NA	NA	NA	NA
	S+C	75	77.6 (74.4-80.8)	39	55.3 (48.4-62.2)	72	55.5 (50.3-60.7)	46	44.7 (39.9-49.6)
	S+R	2	77.3 (49.8-104.7)	1	73.0 (64.0-82.0)	1	64.0 (54.3-73.7)	NA	NA
	C+R	5	58.3 (51.4-65.2)	3	35.1 (16.4-53.8)	NA	NA	NA	NA
	S+C+R	5	79.6 (76.0-83.1)	4	51.9 (33.6-70.3)	5	37.5 (14.7-60.2)	4	32.9 (3.8-62.0)

N – numbers of included studies, SR – survival rate, CI – confidence interval, NA – not available, Combined – two or more styles, GA – gastric adenocarcinoma, SRCC – signet ring cell carcinoma, MC – mucinous adenocarcinoma, Tubular – tubular adenocarcinoma, TNM stage – tumour node metastasis stage, S+C, surgery + chemo; S+R – surgery + radiation, C+R – chemo + radiation, S+C+R – surgery + chemo + radiation, upper – upper one-third of the stomach, middle – middle one-third of the stomach, low – low one-third of the stomach, entire – entire stomach, mixed – two-third or more of the stomach, EGJ – esophagogastric junction

A subgroup analysis on TNM stages (ie, I, II, III, and IV) demonstrated that SRs declined rapidly with an increase in TNM staging. The results of Borrmann classification indicated that patients with early-stage GC had better SRs. Regarding tumour locations, sites in the upper stomach or cardia were associated with lower SRs. Other details of subgroup analyses are shown in **Table 3**.

### Sensitivity analysis

We conducted a sensitivity analysis to assess the effects of specific individual studies on pooled results. Upon removing one study at a time, the pooled SRs remained consistent with the main results (Figures S1-S4 in the **Online Supplementary Document**). The results of our meta-analysis thus appeared to have sufficient stability.

### Publication bias

**Figure 5:**
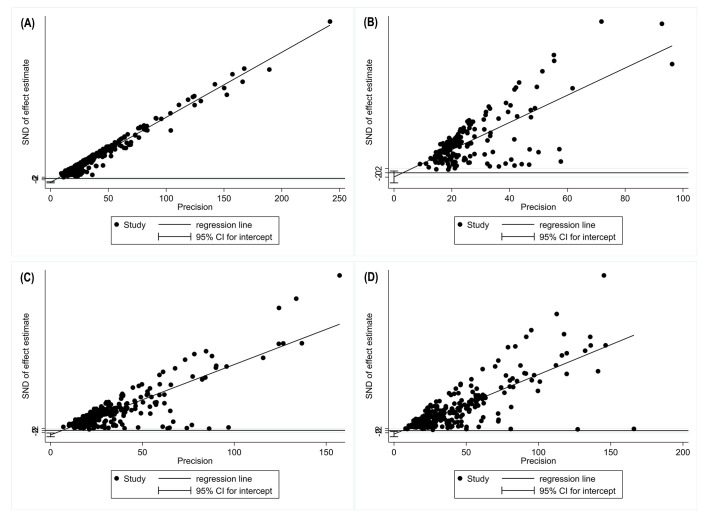
Egger test of publication bias analyses. **Panel A:** one-year survival. **Panel B:** two-year survival. **Panel C:** three-year survival; **Panel B:** five-year survival.

To test the role of publication bias, we carried out a funnel plot analysis and Egger’s test. **Figure 5** and Figure S5 in the **Online Supplementary Document** indicate significant publication bias for the meta-analyses of one-, three-, and five-year SRs.

## DISCUSSION

Our systematic review included 664 original studies, covering 180 798 participants. We observed an increasing trend of long-term survival of GC from 2006 to 2022 and low SRs in GC patients with metastases, including lymph node metastases and distant metastases.

The SRs of GC in our study were much higher than in population-based cancer registries. In the International Cancer Benchmarking Partnership, a large-scale population-based study across seven high-income countries, the age-standardized five-year SRs of GC ranged from 14.0% to 32.8% [22]. Another study combining 17 population-based cancer registries and 659 732 patients in China obtained five-year SRs ranging from 27.4% in 2003-2005 to 35.1% in 2012-2015 [23]. Similar findings have been documented in studies of oesophageal cancer, where hospital-based survival was significantly higher than population-based survival [20]. Population-based cancer statistics are essential for the surveillance of nationwide incidence and mortality. Nevertheless, hospital-based research is an indispensable component of prognostic analysis: it can reveal higher follow-up rates and timely information with respect to disease progression, providing valuable reference data to evaluate anti-cancer treatment.

We observed a rising trend in long-term SRs of GC since 2000. The substantial improvement in hospital-based survival is likely due to progress in medical technology. Consistent with our findings, research has indicated a persistent decline in mortality from GC in China [7] and a steady growth in SRs for the disease in Europe and the United States [24-26]. Notably, inconsistent with the abovementioned results, one-year survival declined between 2006 and 2022 while two-year survival fell from 2011 to 2022. The two-year SR was also significantly lower than three- and five-year SRs between 2016 and 2022. These abnormalities were the result of the large number of original studies focusing on short-term survival (ie, one and two years) of advanced GC in recent years. The prognosis was especially poor for patients with advanced GC, with one- and two-year SRs being demonstrably lower.

In our subgroup analysis, studies of GA accounted for more than 90% of all eligible studies. The pooled SRs of GA ranged from 45.0% (95% CI = 40.0%-50.0%) at five years to 71.7% (95% CI = 68.0%-75.4%) at one year. A small set of studies reported the Laurén classification in our meta-analysis. The TNM classification system is crucial for making clinical decisions and prognostic estimations [27]. Our findings showed a sizeable drop in SRs, accompanied by an increase in TNM stages, with a five-year survival of 83.9% for Stage I and 9.1% for Stage IV. The Borrmann classification system has been commonly adopted in advanced GC studies and is frequently applied to describe clinical features [28,29]. Our pooled analysis showed that SRs fell as Borrmann types increased, exemplifying the performance of this classification protocol in predicting GC prognosis. Scholars have similarly reported that proximal GC has a relatively poor prognosis [30]. We observed lower SRs in patients with carcinoma in the upper stomach than in the middle or lower stomach, which was consistent with the literature.

We identified higher SRs in areas with high GC prevalence (including Shandong, Fujian, Liaoning, Gansu, and Jiangsu) [15,31-33]. These areas likely devote more effort to clinical intervention and screening for GC, leading to a better prognosis [15]. These provinces also have relatively higher socioeconomic conditions within China, which can facilitate access to early clinical intervention. The prognosis of GC may therefore improve despite the high incidence of GC remaining uncontrolled. Additionally, the high detection rate of early GC in these high-risk regions (up to 47.2%-67%) results in relatively lower-case fatality rates [34]. However, while Japan and Korea do have a nationwide screening programme, China does not [14,35].

First-line medication approaches identified in this meta-analysis were surgical. New technologies (eg, endoscopic mucosal resections and laparoscopic and robotic gastrectomy) and therapies (eg, neoadjuvant and perioperative chemotherapy, preoperative or postoperative radiation therapy, and postoperative adjuvant chemotherapy) have improved GC prognosis [5,8,36]. We found that patients who underwent surgery had better SRs; patients who received both surgery and chemotherapy and radiation had poorer SRs, potentially attributed to their disease severity. Regarding gender differences, male patients’ SR was 2.19 times higher than for females, coincident with previous findings [1,37].

As a nationwide systematic review, the representability and reliability of our findings might be substantially impacted by the methodology qualities of included studies. After the exclusion of 12 original studies that did not pass the quality assessment, this study included 664 eligible studies that reported survival of GC.

Our study has several limitations that are often present in systematic reviews and meta-analyses on analogous topics. First, given that the included studies spanned the period from 2000 to 2022, GC classification approaches varied. Second, as our study focused on GC survival in hospital-based studies, significant publication bias might be introduced in the pooled results. Third, the included studies were highly heterogenous, possibly because of how clinical information was gathered, chosen follow-up methods, and patient diversity. Finally, even though we included original studies conducted in hospitals in China, we could not exclude the foreigners who were hospitalized in Chinese hospitals.

## CONCLUSIONS

The long-term survival of GC improved since 2000. Patients with metastasis, those at advanced TNM stages, and those with gastric stump cancer and cancer in the upper stomach had particularly poor prognoses. Higher SRs corresponded to hospital-based studies rather than population-based surveillance data sets. This indicates that accessible health care provided by hospitals can effectively improve the survival of GC.

## Additional material


Online Supplementary Document
