# Supplementary material for: Survival of gastric cancer in China from 2000 to 2022: A nationwide systematic review of hospital-based studies
Source: J Glob Health. 2022 Dec 17;12:11014. doi: 10.7189/jogh.12.11014 (PMC9759711; doi:10.7189/jogh.12.11014)
Supplement: Online Supplementary Document [file jogh-12-11014-s001.pdf]

ONLINE SUPPLEMENTARY DOCUMENT

Title: Survival of gastric cancer in China from 2000 to 2022: A nationwide systematic review of hospital-based studies

Authors: Houqiang Li, Han Zhang, Hujia Zhang, Youxin Wang, Xiaobing Wang, Haifeng Hou

Table S1 Checklist of PRISMA Statement

Section/Topic	Checklist Item	Reported or not
TITLE		
Title	Identify the report as a systematic review, meta-analysis, or both.	Y
ABSTRACT		
Structured summary	Provide a structured summary including, as applicable: background; objectives; data sources; study eligibility criteria, participants, and interventions; study appraisal and synthesis methods; results; limitations; conclusions and implications of key findings; systematic review registration number.	Y
INTRODUCTION		
Rationale	Describe the rationale for the review in the context of what is already known.	Y
Objectives	Provide an explicit statement of questions being addressed with reference to participants, interventions, comparisons, outcomes, and study design (PICOS).	Y
METHODS		
Protocol and registration	Indicate if a review protocol exists, if and where it can be accessed (e.g., Web address), and, if available, provide registration information including registration number.	Y
Eligibility criteria	Specify study characteristics (e.g., PICOS, length of follow-up) and report characteristics (e.g., years considered, language, publication status) used as criteria for eligibility, giving rationale.	Y
Information sources	Describe all information sources (e.g., databases with dates of coverage, contact with study authors to identify additional studies) in the search and date last searched.	Y
Search	Present full electronic search strategy for at least one database, including any limits used, such that it could be repeated.	Y
Study selection	State the process for selecting studies (i.e., screening, eligibility, included in systematic review, and, if applicable, included in the	Y

	meta-analysis).	
Data collection process	Describe method of data extraction from reports (e.g., piloted forms, independently, in duplicate) and any processes for obtaining and confirming data from investigators.	Y
Data items	List and define all variables for which data were sought (e.g., PICOS, funding sources) and any assumptions and simplifications made.	Y
Risk of bias in individual studies	Describe methods used for assessing risk of bias of individual studies (including specification of whether this was done at the study or outcome level), and how this information is to be used in any data synthesis.	Y
Summary measures	State the principal summary measures (e.g., risk ratio, difference in means).	Y
Synthesis of results	Describe the methods of handling data and combining results of studies, if done, including measures of consistency (e.g., I2) for each meta-analysis.	Y
Risk of bias across studies	Specify any assessment of risk of bias that may affect the cumulative evidence (e.g., publication bias, selective reporting within studies).	Y
Additional analyses	Describe methods of additional analyses (e.g., sensitivity or subgroup analyses, meta-regression), if done, indicating which were pre-specified.	Y
RESULTS		
Study selection	Give numbers of studies screened, assessed for eligibility, and included in the review, with reasons for exclusions at each stage, ideally with a flow diagram.	Y
Study characteristics	For each study, present characteristics for which data were extracted (e.g., study size, PICOS, follow-up period) and provide the citations.	Y
Risk of bias within studies	Present data on risk of bias of each study and, if available, any outcome-level assessment (see Item 12).	Y
Results of individual studies	For all outcomes considered (benefits or harms), present, for each study: (a) simple summary data for each intervention group and (b) effect estimates and confidence intervals, ideally with a forest plot.	Y
Synthesis of results	Present results of each meta-analysis done, including confidence intervals and measures of consistency.	Y
Risk of bias across studies	Present results of any assessment of risk of bias across studies (see Item 15).	Y
Additional analysis	Give results of additional analyses, if done (e.g., sensitivity or subgroup analyses, meta-regression [see Item 16]).	Y
DISCUSSION		Y
Summary of evidence	Summarize the main findings including the strength of evidence for each main outcome; consider their relevance to key groups (e.g., health care providers, users, and policy makers).	Y

Limitations	Discuss limitations at study and outcome level (e.g., risk of bias), and at review level (e.g., incomplete retrieval of identified research, reporting bias).	Y
Conclusions	Provide a general interpretation of the results in the context of other evidence, and implications for future research.	Y
FUNDING		
Funding	Describe sources of funding for the systematic review and other support (e.g., supply of data)	Y

Y: the item was reported in article, N: the item was not reported.

Table S2 Search strategy of systematic review

Databases		Search strategy	Numbers of studies
**English databases**			
	PubMed	((stomach neoplasms]) OR (gastric cancer) OR (gastric carcinoma) AND ((China) OR (Chinese)) AND (hospital) AND (survival)	10,175
	Embase	('stomach neoplasms' OR 'stomach neoplasms' OR 'gastric cancer' OR 'gastric carcinoma') AND ('China' OR 'Chinese’) AND ('hospital') AND ('survival' OR 'survival')	12,443
	Web of science	(((stomach neoplasms) OR (gastric cancer) OR (gastric carcinoma)) AND ((China) OR (Chinese)) AND (survival) AND (hospital))	9,980
**Chinese databases**			
	CNKI	胃癌(gastric cancer) AND 生存(survival) AND 医院(hospital)	2,271
	Wanfang	胃癌(gastric cancer) AND 生存(survival) AND 医院(hospital)	1,820

Table S3 Scale for quality assessment

Criteria	Score
**Representativeness of cases**	
Characteristics of participants were described.	1
Consecutive/randomly selected from case population was clearly defined.	1
Eligible patients are similar to controls, in term of age, gender and other important characteristics.	1
The percentage of loss to follow-up was provided, or the reasons of loss to follow-up were mentioned.	1
**Accuracy of information**	
Methods of variable measurement were offered	1
Definitions of outcome were offered.	1
**Statistical analyses**	
Methods of statistical analyses were adequate to resolve research hypothesis.	1
Multivariate analyses were performed.	1
**Final question**	
If there were any other important flaws in the design, the study would be not included.	1

Table S4 Characteristics of included studies

No	Author	Publication year	Areas	Design	Quality score	Age	Sample size	Male	Female	Language
1	Wu AW	2010	Beijing	R	6	58.8	2312	1633	679	English
2	Li C	2010	Shanghai	R	7	58.1	128	58	70	English
3	Qiu MZ	2011	Guangdong	R	7	NA	1000	683	317	English
4	Ye YW	2011	Shanghai	R	8	49.7	162	89	73	English
5	Zhang H	2011	Liaoning	R	6	NA	1604	1172	432	English
6	Li P	2012	Zhejiang	R	5	53	46	35	11	English
7	Li X	2013	Tianjin	R	7	54	162	90	72	English
8	Kong FM	2013	Tianjin	R	7	60.2	1296	966	330	English
9	Zhu Z	2014	Liaoning	R	7	NA	932	669	263	English
10	Zeng WJ	2014	Hubei	R	7	NA	533	389	144	English
11	Zhang WH	2014	Sichuan	R	7	56.8	1654	1067	587	English
12	Wang J	2014	Jiangsu	R	6	NA	605	438	167	English
13	Zhang JK	2014	Liaoning	R	7	NA	830	444	386	English
14	Sun XR	2015	Liaoning	R	7	59	265	194	71	English
15	Zheng XZ	2015	Jiangsu	R	6	NA	60	NA	NA	English
16	Wang W	2015	Guangdong&Fujian	R	7	57.8	5327	3856	1471	English
17	Tang Z	2015	Zhejiang	R	6	59.8	165	124	41	English
18	Yang K	2016	Sichuan	R	7	NA	1365	409	956	English
19	Sun HL	2017	Jiangsu	R	7	65.5	778	572	206	English
20	Anup S	2017	Fujian	R	7	57.2	132	94	38	English
21	Wang JF	2018	Hebei	R	7	63	128	99	29	English
22	Xue WJ	2018	Shanghai	R	7	63	197	129	68	English
23	Hu M	2018	Sichuan	R	6	NA	164	121	43	English
24	Liu DH	2018	Heilongjiang	R	7	57	122	88	34	English
25	Gao YH	2019	Beijing	R	6	59.7	502	322	180	English
26	Shu P	2019	Jiangsu	R	6	58.5	489	358	131	English
27	Liang Y	2019	Tianjin&Hainan	R	7	NA	2344	1667	677	English
28	Zhai Z	2020	Heilongjiang	R	7	58.6	4744	3502	1242	English
29	Lei T	2020	Hunan	R	6	55.3	177	92	85	English
30	Sheng LL	2020	Anhui	R	6	60.2	299	205	94	English
31	Liu S	2020	Beijing	R	7	61.9	209	180	29	English
32	Tan YE	2020	Liaoning	R	6	NA	3520	2566	954	English
33	Huang X	2021	Guangxi	P	6	57.7	161	104	57	English
34	Zhou LY	2021	Henan	R	6	NA	99	NA	NA	English
35	Ren B	2000	Zhejiang	R	5	NA	52	33	19	Chinese
36	Qi HL	2001	Henan	R	5	NA	62	49	13	Chinese
37	Li Q	2001	Tianjin	R	7	48.9	81	NA	NA	Chinese
38	Shi H	2002	Anhui	R	8	55.4	281	198	83	Chinese
39	Huang J	2003	Guangdong	R	5	NA	26	19	7	Chinese

40	Che XM	2003	Shaanxi	R	6	54.1	29	4	25	Chinese
41	Fei BJ	2003	Jiangsu	R	7	56.7	890	593	297	Chinese
42	Yang MD	2004	heilongjiang	R	5	58	63	41	22	Chinese
43	Guo H	2004	Henan	R	5	NA	40	32	8	Chinese
44	Shi Y	2004	Jilin	P	6	60.7	162	140	22	Chinese
45	Li JW	2004	Tianjin	R	6	NA	814	605	209	Chinese
46	Chen DL	2004	Anhui	R	6	NA	80	61	19	Chinese
47	Lin G	2005	Fujian	R	6	NA	79	48	31	Chinese
48	Yang WM	2005	Guangdong	R	5	NA	46	NA	NA	Chinese
49	Chen SY	2005	Shanghai	R	6	56	96	59	37	Chinese
50	Yang QB	2005	Liaoning	R	6	55.3	409	312	97	Chinese
51	Zhang YQ	2005	Liaoning	R	6	NA	209	NA	NA	Chinese
52	Wang TM	2005	Anhui	R	6	NA	53	NA	NA	Chinese
53	Chen BC	2005	Hunan	P	5	51.7	110	72	38	Chinese
54	Xu YP	2005	Hebei	R	6	NA	80	61	19	Chinese
55	Li WS	2006	Liaoning	R	7	54.9	51	37	14	Chinese
56	Wang YZ	2006	Fujian	R	7	NA	98	NA	NA	Chinese
57	Li YJ	2006	Jilin	R	6	57	60	44	16	Chinese
58	Quan XN	2006	Jilin	R	6	57.5	50	42	8	Chinese
59	Liu B	2007	Liaoning	R	7	56.3	45	35	10	Chinese
60	Liu WS	2007	Shaanxi	R	5	NA	97	NA	NA	Chinese
61	Feng B	2007	Shandong	P	5	NA	50	24	26	Chinese
62	Wang TM	2007	Anhui	R	5	NA	65	NA	NA	Chinese
63	Jiang YM	2007	Guangdong	R	5	67.2	102	86	16	Chinese
64	Xu Y	2007	Shanghai	R	7	NA	749	601	148	Chinese
65	Wu DB	2008	Guangdong	R	6	NA	57	NA	NA	Chinese
66	Wan H	2008	Jiangsu	P	5	NA	72	NA	NA	Chinese
67	Liu SL	2008	Shandong	P	6	NA	65	36	29	Chinese
68	Zhang Y	2008	Liaoning	R	6	NA	386	287	99	Chinese
69	Zhang L	2008	Shaanxi	R	6	50.5	428	329	99	Chinese
70	You Z	2008	Tianjin	R	5	NA	69	NA	NA	Chinese
71	Song XF	2008	Liaoning	R	6	56.5	263	176	87	Chinese
72	Wu WQ	2008	Zhejiang	R	6	51.3	84	43	41	Chinese
73	Zhang Y	2008	ZheJiang	R	6	54.6	63	40	23	Chinese
74	Lin T	2009	Guangdong	R	6	NA	122	NA	NA	Chinese
75	Zhou LX	2009	Zhejiang	R	6	NA	81	62	19	Chinese
76	Huang YH	2009	Yunnan	R	5	NA	55	NA	NA	Chinese
77	Liu DF	2009	Guangdong	R	5	NA	407	NA	NA	Chinese
78	Luo Y	2009	Jiangsu	R	5	NA	39	NA	NA	Chinese
79	Xu XY	2009	Anhui	R	6	NA	115	NA	NA	Chinese
80	Wang C	2009	Shanghai	R	6	56.2	459	305	154	Chinese
81	Bai XF	2009	Beijing	P	7	62	97	50	47	Chinese

82	Liu FL	2009	Shanghai	P	6	NA	148	93	55	Chinese
83	Ding XW	2009	Tianjin	P	6	NA	276	204	72	Chinese
84	Xie YQ	2009	Beijing	R	6	NA	119	88	31	Chinese
85	Jia ZS	2009	Shandong	R	6	56.5	806	596	210	Chinese
86	Mei D	2009	Liaoning	R	7	58.3	791	573	218	Chinese
87	Xiao ZL	2009	Shanghai	R	6	NA	78	52	26	Chinese
88	Hong HJ	2009	Guangdong	R	6	67.2	79	62	17	Chinese
89	Xu DZ	2009	Guangdong	R	7	60.2	906	609	297	Chinese
90	Cai AZ	2010	Beijing	R	6	NA	121	NA	NA	Chinese
91	Lin L	2010	Fujian	R	5	NA	36	NA	NA	Chinese
92	Cao ND	2010	Shanghai	P	5	NA	88	NA	NA	Chinese
93	Zhang H	2010	Liaoning	R	7	NA	256	NA	NA	Chinese
94	Yu SF	2010	Zhejiang	R	7	NA	140	NA	NA	Chinese
95	Zhang ZJ	2010	Zhejiang	R	5	55.6	90	63	27	Chinese
96	Huang WL	2010	Jilin	R	5	69.5	40	31	9	Chinese
97	Wang DY	2010	Liaoning	R	5	NA	42	NA	NA	Chinese
98	Xue YW	2010	heilongjiang	R	7	NA	630	480	150	Chinese
99	Li XB	2010	Shaanxi	R	7	NA	934	NA	NA	Chinese
100	Li SY	2010	Beijing	R	7	NA	45	26	19	Chinese
101	Jiao ZK	2010	Hebei	R	6	56.7	161	118	43	Chinese
102	Chen J	2010	Shanghai	R	6	NA	431	316	115	Chinese
103	Chen JH	2010	Guangdong	R	7	NA	419	283	136	Chinese
104	Li XB	2010	Shaanxi	R	7	NA	934	NA	NA	Chinese
105	Shi XR	2011	Hubei	R	6	46.5	93	61	32	Chinese
106	Zhao L	2011	Shandong	R	7	NA	86	NA	NA	Chinese
107	Li L	2011	Hebei	R	5	NA	194	NA	NA	Chinese
108	Peng G	2011	Jiangxi	P	6	NA	133	75	58	Chinese
109	Chen PZ	2011	Jiangsu	R	5	NA	20	10	10	Chinese
110	Li WH	2011	Liaoning	R	6	NA	146	NA	NA	Chinese
111	Huang F	2011	Fujian	R	7	56.3	112	68	44	Chinese
112	Ma C	2011	Henan	R	5	62.5	24	17	7	Chinese
113	Tian BC	2011	Shanxi	R	5	NA	12	NA	NA	Chinese
114	Ning ZF	2011	Hubei	R	5	61.6	66	39	27	Chinese
115	Meng LF	2011	Zhejiang	R	5	NA	32	NA	NA	Chinese
116	Luo CY	2011	Guangdong	P	6	65.3	174	121	53	Chinese
117	Zhang JF	2011	Henan	R	6	53.7	120	67	53	Chinese
118	Lin HD	2011	Guangxi	R	5	NA	53	NA	NA	Chinese
119	Zhou B	2011	Hunan	R	5	56.3	116	86	30	Chinese
120	Yang M	2011	heilongjiang	R	5	NA	946	NA	NA	Chinese
121	Shen CL	2011	Shanghai	R	6	NA	135	89	46	Chinese
122	Yu ZY	2011	Shandong	R	5	NA	121	NA	NA	Chinese
123	Yu YS	2011	Jiangsu	P	6	53.8	74	46	28	Chinese

124	Zhang ML	2011	Jiangsu	R	7	NA	489	359	130	Chinese
125	Yang ZJ	2011	Hebei	R	7	NA	80	52	28	Chinese
126	Zhu GY	2011	Zhejiang	R	5	NA	206	142	64	Chinese
127	Ma XM	2011	Liaoning	R	6	NA	843	NA	NA	Chinese
128	Jiang ZH	2012	Jiangxi	P	6	51.2	60	39	21	Chinese
129	Guo YQ	2012	Liaoning	R	7	NA	550	NA	NA	Chinese
130	Wang J	2012	Shanghai	R	7	NA	606	406	200	Chinese
131	Wang B	2012	Jiangsu	R	7	64	351	252	99	Chinese
132	Ge YP	2012	Beijing	P	6	NA	44	30	14	Chinese
133	Wang JL	2012	Liaoning	P	5	64.4	64	41	23	Chinese
134	Wu J	2012	Jiangsu	P	6	NA	80	NA	NA	Chinese
135	Xu X	2012	Jiangxi	R	6	NA	68	NA	NA	Chinese
136	Lin Z	2012	Guangxi	P	7	NA	80	49	31	Chinese
137	Liu R	2012	Beijing	R	6	NA	88	67	21	Chinese
138	Wu CF	2012	Henan	R	6	NA	260	NA	NA	Chinese
139	Liu JZ	2012	Shanghai	R	6	61.7	153	97	56	Chinese
140	Liu SW	2012	Hebei	R	6	NA	387	333	54	Chinese
141	Zhao YZ	2012	Henan	R	6	NA	193	NA	NA	Chinese
142	Guo CY	2013	Shaanxi	R	6	58.6	66	50	16	Chinese
143	Hong LQ	2013	Fujian	R	6	59.2	32	25	7	Chinese
144	Hu JH	2013	Henan	P	7	NA	84	NA	NA	Chinese
145	Lai JJ	2013	Guangdong	R	5	NA	104	63	41	Chinese
146	Zhang LT	2013	Anhui	P	6	NA	82	39	43	Chinese
147	Zhang WN	2013	Shaanxi	R	5	NA	56	32	24	Chinese
148	Zhang YF	2013	Guangxi	P	5	NA	86	59	27	Chinese
149	He YJ	2013	Hunan	R	7	NA	89	53	36	Chinese
150	Lin T	2013	Shaanxi	R	6	NA	200	141	59	Chinese
151	Du BB	2013	Liaoning	R	6	60.6	112	69	43	Chinese
152	Lei Y	2013	Beijing	R	5	58.5	75	54	21	Chinese
153	Ma YH	2013	Xinjiang	R	7	NA	164	129	35	Chinese
154	Li Y	2013	Guangdong	R	6	65.1	41	37	4	Chinese
155	Gong HJ	2013	Shanghai	R	7	60.2	151	105	46	Chinese
156	Shan LQ	2013	Jiangsu	R	7	NA	1451	996	455	Chinese
157	Huang JQ	2014	Guangdong	R	6	57.1	85	47	38	Chinese
158	Chen w	2014	Zhejiang	R	5	NA	21	NA	NA	Chinese
159	Wang ZY	2014	Jiangxi	R	7	NA	126	NA	NA	Chinese
160	Zhang CL	2014	Henan	R	5	NA	12	NA	NA	Chinese
161	Su Y	2014	Jiangxi	P	6	NA	120	64	56	Chinese
162	Li Y;	2014	Shanghai	R	6	NA	209	NA	NA	Chinese
163	He XW	2014	Zhejiang	P	6	NA	60	38	22	Chinese
164	Tang SZ	2014	Hunan	R	6	NA	62	43	19	Chinese
165	Li S;	2014	Henan	P	7	NA	166	87	79	Chinese

166	Huang FS	2014	Guangxi	R	6	68.2	38	22	16	Chinese
167	Shi YJ	2014	Guangdong	R	5	59.7	35	18	17	Chinese
168	Geng XW	2014	Hunan	R	7	47.9	94	61	33	Chinese
169	Ren ZJ	2014	Hunan	R	6	NA	50	33	17	Chinese
170	Zhao DY	2014	Tianjin	R	6	65.7	60	46	14	Chinese
171	Yan L	2014	Henan	P	7	NA	86	NA	NA	Chinese
172	Li MZ	2014	Hubei	P	6	NA	92	54	38	Chinese
173	Dong L	2014	Jiangxi	R	6	NA	76	NA	NA	Chinese
174	Chai JH	2014	Henan	R	6	NA	300	218	82	Chinese
175	Zhang J	2014	Henan	R	6	NA	143	91	52	Chinese
176	Li SZ	2014	Jilin	R	6	61	108	78	30	Chinese
177	Huang ZQ	2014	Fujian	R	7	NA	216	137	79	Chinese
178	Zhang C	2014	Ningxia	P	6	NA	57	41	16	Chinese
179	Wei YL	2014	Sichuan	P	6	41	60	31	29	Chinese
180	Liu SP	2015	Hainan	P	7	48.7	160	108	52	Chinese
181	Zhou H	2015	Shandong	P	6	NA	52	NA	NA	Chinese
182	Zhang W	2015	Beijing	R	7	NA	130	NA	NA	Chinese
183	Wang LJ	2015	Beijing	R	6	NA	178	127	51	Chinese
184	Kang Y	2015	Shanxi	P	6	58.7	160	98	62	Chinese
185	Zhao Y	2015	Sichuan	R	5	NA	38	NA	NA	Chinese
186	Qiao SF	2015	Liaoning	R	7	NA	86	67	19	Chinese
187	Wang YF	2015	Hebei	P	6	59	207	158	49	Chinese
188	Zhong JC	2015	Jiangsu	P	6	NA	60	40	20	Chinese
189	Tian L	2015	Shanghai	R	6	NA	85	69	16	Chinese
190	Lu G	2015	Beijing	P	6	NA	72	43	29	Chinese
191	Luo ZM	2015	Henan	P	7	NA	161	NA	NA	Chinese
192	Wang Z	2015	Jiangxi	P	7	43.5	80	54	26	Chinese
193	Yang XA	2015	Henan	R	5	74	36	28	8	Chinese
194	Di YC	2015	Henan	P	6	45.7	60	37	23	Chinese
195	Liu RQ	2015	Fujian	P	5	49.2	167	96	71	Chinese
196	Shen NY	2015	Shaanxi	P	7	NA	140	64	76	Chinese
197	Jin J	2015	Hebei	P	6	NA	80	47	33	Chinese
198	Ni SM	2015	Zhejiang	P	6	NA	66	43	23	Chinese
199	Zhou N	2015	Xinjiang	P	7	NA	60	34	26	Chinese
200	Liu YP	2015	Hunan	P	7	NA	120	59	61	Chinese
201	Zhu HT	2015	Liaoning	R	7	NA	106	74	32	Chinese
202	Hou Z	2015	Liaoning	P	6	NA	50	31	19	Chinese
203	Dong SJ	2015	Hebei	P	7	NA	128	75	53	Chinese
204	Peng JJ	2015	Guangdong	R	7	57	942	650	292	Chinese
205	Cui JG	2015	Tianjin	R	7	NA	300	217	83	Chinese
206	Zhang Y	2015	Shandong	P	7	NA	76	NA	NA	Chinese
207	Zhang S	2015	Beijing	R	6	NA	504	387	117	Chinese

208	Zhong HQ	2015	Jiangxi	R	6	NA	97	65	32	Chinese
209	Liang XY	2016	HeiLongJiang	P	6	72.9	86	59	27	Chinese
210	Zhou X	2016	Sichuan	P	7	54	186	102	84	Chinese
211	Zhang XT	2016	Shandong	P	7	NA	126	78	48	Chinese
212	Wu B	2018	Ningxia	R	6	30.5	368	255	113	Chinese
213	Jian JL	2019	Fujian	R	6	59	984	704	280	Chinese
214	Zhan YB	2019	Shanghai	P	5	63.8	106	59	47	Chinese
215	Sun JG	2019	Shanxi&Shaanxi	P	6	56.5	60	36	24	Chinese
216	Jiao JG	2019	Shandong	P	6	NA	95	NA	NA	Chinese
217	Zhou H	2019	Jiangsu	P	6	51.8	60	36	24	Chinese
218	Zhang K	2019	Hunan	R	6	45.8	84	46	38	Chinese
219	Zhen Y	2019	Fujian	P	7	NA	101	NA	NA	Chinese
220	Qin RF	2019	Hebei	P	6	58.3	80	49	31	Chinese
221	Mo L	2019	Guangxi	P	6	63.4	100	57	43	Chinese
222	Zhang ZH	2019	Hubei	P	7	49.9	76	56	20	Chinese
223	Li ZH	2019	Zhejiang	R	5	63.2	100	59	41	Chinese
224	Dai B	2019	Henan	P	7	62.4	109	52	57	Chinese
225	Shi Y	2019	Anhui	P	6	58.4	68	39	27	Chinese
226	Wu K	2019	Guangdong	P	7	63.8	88	60	28	Chinese
227	Xiao Y	2019	Shandong	P	7	NA	102	47	55	Chinese
228	Qiu F	2019	Sichuan	R	6	56.5	81	45	36	Chinese
229	Qiao B	2019	Sichuan	P	5	58.1	62	38	24	Chinese
230	Qi XY	2019	Anhui	P	7	NA	284	211	73	Chinese
231	Zhang GL	2019	Hubei	P	6	59.8	70	45	25	Chinese
232	Xu Y	2019	Anhui	P	5	55.8	120	74	46	Chinese
233	Zhang GZ	2019	Anhui	P	7	46	78	46	32	Chinese
234	Zhao GC	2019	Jilin	P	7	56.9	100	62	38	Chinese
235	Chen ZH	2019	Guangxi	P	7	NA	90	49	41	Chinese
236	Sun X;	2019	Anhui	R	6	NA	286	192	94	Chinese
237	Wang G;	2019	Qinghai	R	6	61.6	102	88	14	Chinese
238	Gao P	2019	Jiangxi	R	7	NA	101	68	33	Chinese
239	Zhang P	2019	Henan	R	6	NA	231	132	99	Chinese
240	Chen J;	2019	Shaanxi	R	6	58.3	1973	1478	495	Chinese
241	Jia YX	2019	Henan	R	6	NA	107	72	35	Chinese
242	Mai MT	2019	Xinjiang	R	7	NA	118	93	25	Chinese
243	Li YL	2019	Fujian	R	6	NA	341	NA	NA	Chinese
244	Bao GJ	2019	Shanghai	P	6	56.2	102	72	30	Chinese
245	Long M	2019	Jiangxi	R	6	NA	62	31	31	Chinese
246	Song JF	2019	Hainan	P	6	NA	93	70	23	Chinese
247	Liu JF	2019	Hebei	R	6	NA	57	NA	NA	Chinese
248	Wang X;	2019	Shanxi	R	6	NA	236	138	98	Chinese
249	Xu Y	2019	Beijing	R	6	NA	120	65	55	Chinese

250	Zhang Y	2019	Gansu	R	6	NA	63	43	20	Chinese
251	Bu LB	2019	Xinjiang	P	6	NA	80	51	29	Chinese
252	Ye ZJ	2019	Guangdong	R	7	69	200	139	61	Chinese
253	Wu YC	2019	Beijing	R	7	NA	178	122	56	Chinese
254	Ji ZH	2019	Beijing	R	7	NA	115	56	59	Chinese
255	Wang ZH	2019	Sichuan	R	6	NA	70	NA	NA	Chinese
256	Guo LY	2019	Hebei	R	6	NA	157	130	27	Chinese
257	Zhang MM	2019	Shandong	R	6	NA	78	55	23	Chinese
258	Wang RH	2019	Xinjiang	R	6	NA	196	139	57	Chinese
259	Zhang CY	2019	Guizhou	R	6	NA	86	67	19	Chinese
260	Chen JJ	2019	Jiangsu	R	6	NA	50	30	20	Chinese
261	He YX	2019	Shaanxi	R	6	58.4	667	515	152	Chinese
262	Nie HY	2019	Liaoning	R	7	NA	153	NA	NA	Chinese
263	Chen Q;	2019	Fujian	R	5	45	40	NA	NA	Chinese
264	Zhang YY	2019	Zhejiang	R	6	NA	194	137	57	Chinese
265	Zheng JY	2019	Fujian	R	6	57.4	25	19	6	Chinese
266	Liu YN	2019	Henan	R	6	59.2	210	145	65	Chinese
267	Hou JP	2019	Henan	R	7	NA	84	49	34	Chinese
268	Dai XC	2019	Jiangsu	P	6	NA	86	56	30	Chinese
269	Li H;	2019	Zhejiang	R	7	NA	80	NA	NA	Chinese
270	Chen W;	2019	Jiangsu	R	6	NA	113	72	41	Chinese
271	Bao Y;	2019	Anhui	R	6	NA	97	57	40	Chinese
272	Shen YF	2019	Jiangsu	R	6	NA	49	32	17	Chinese
273	Zou ZL	2019	Jiangsu	P	6	63.9	60	37	23	Chinese
274	Li TJ	2019	Yunnan	R	5	NA	42	20	22	Chinese
275	Wang KW	2019	Guangdong	R	6	55.8	402	276	126	Chinese
276	Yang LP	2020	Gansu	R	7	57	107	61	46	Chinese
277	Ming R	2020	Sichuan	P	7	NA	68	52	16	Chinese
278	Wang Y	2020	Shaanxi	P	7	NA	110	70	40	Chinese
279	Feng Y	2020	Guangdong	R	5	58.1	53	31	22	Chinese
280	Gong YB	2020	Liaoning	R	7	NA	2522	1846	676	Chinese
281	Zhang W	2020	Beijing	R	8	NA	52	NA	NA	Chinese
282	Guo XD	2020	Shanghai	P	5	NA	77	47	30	Chinese
283	Xu QL	2020	Shaanxi	P	6	59.8	110	64	46	Chinese
284	Ding PA	2020	Hebei	P	7	NA	225	162	63	Chinese
285	Gu JB	2020	Henan	P	7	62	98	64	34	Chinese
286	Zhan HJ	2020	Tianjin	R	6	59.5	170	90	80	Chinese
287	Zhang WH	2020	Sichuan	P	8	NA	4516	3183	1333	Chinese
288	Liang WQ	2020	Beijing	R	5	NA	3431	NA	NA	Chinese
289	Guo J	2020	Hebei	P	5	65	60	31	29	Chinese
290	Sun YC	2020	Henan	P	7	60	86	47	39	Chinese
291	Ding PA	2020	Hebei	R	7	NA	476	NA	NA	Chinese

292	Sheng LL	2020	Anhui	P	5	NA	216	118	98	Chinese
293	Zhai Z	2020	Heilongjiang	R	7	NA	625	433	192	Chinese
294	Sha JP	2020	Henan	P	7	NA	116	78	38	Chinese
295	Ding PA	2020	Hebei	R	7	NA	104	NA	NA	Chinese
296	Dong TT	2020	Jiangsu	R	7	62.3	391	112	279	Chinese
297	Han WF	2020	Gansu	R	7	71	216	166	50	Chinese
298	Han BL	2020	Heilongjiang	R	7	NA	891	649	242	Chinese
299	Liu XC	2020	Beijing	R	5	59.8	60	47	13	Chinese
300	Ding PA	2020	Hebei	R	7	NA	2273	NA	NA	Chinese
301	Zhao XL	2020	Beijing	P	6	NA	116	NA	NA	Chinese
302	Zhu Y	2020	Hubei	R	7	NA	390	NA	NA	Chinese
303	Wu YH	2020	Jiangsu	R	7	NA	72	55	17	Chinese
304	Han T	2020	Tianjin	P	5	57.7	481	314	167	Chinese
305	Wu J	2020	Anhui	P	7	NA	98	NA	NA	Chinese
306	Zhou M	2020	Hebei	R	6	56.3	118	83	35	Chinese
307	Zhang XL	2020	Sichuan	R	6	NA	120	72	48	Chinese
308	Yin F	2020	Yunnan	R	6	59.8	157	103	54	Chinese
309	Yi YJ	2020	Sichuan	P	7	58.3	103	49	54	Chinese
310	Zhu ZY	2020	Heilongjiang	R	7	NA	645	418	227	Chinese
311	Jiang TP	2020	Anhui	P	6	50.2	200	123	77	Chinese
312	Yang JQ	2020	Chongqing	P	6	64.7	500	274	226	Chinese
313	Peng M	2020	Sichuan	P	6	54.4	78	44	34	Chinese
314	Guo J	2020	Henan	P	6	60.7	101	78	23	Chinese
315	Zhang XP	2020	Shandong	P	7	59.2	597	342	255	Chinese
316	Sun HX	2020	Sichuan	P	6	NA	128	NA	NA	Chinese
317	Chen L	2020	Hunan	P	6	NA	105	NA	NA	Chinese
318	Qian XL	2020	Jiangsu	P	7	NA	102	79	23	Chinese
319	Zheng T	2020	Hebei	P	6	57.1	48	35	13	Chinese
320	Liu JT	2020	Jiangsu	R	6	63.5	713	531	182	Chinese
321	Pan H	2020	Guizhou	R	6	NA	231	150	81	Chinese
322	Zhu QT	2020	Zhejiang	R	6	NA	245	176	69	Chinese
323	Guo JB	2020	Jiangsu	R	6	65	160	120	40	Chinese
324	Shi YC	2020	Shaanxi	P	7	NA	432	347	85	Chinese
325	Duan ZX	2020	Henan	R	6	NA	68	56	12	Chinese
326	LU GJ	2020	Shandong	P	6	NA	528	359	169	Chinese
327	Wang H	2020	Zhejiang	P	7	NA	466	327	139	Chinese
328	Zhang QR	2020	Beijing	R	6	59.3	122	75	47	Chinese
329	Wu JF	2020	Zhejiang	R	6	62.4	87	63	24	Chinese
330	Tan J	2020	Shaanxi	R	6	NA	623	NA	NA	Chinese
331	Hao K	2020	Shandong	P	6	57.4	368	187	181	Chinese
332	Shen S	2020	Shandong	P	6	58.9	566	419	147	Chinese
333	Li Y	2020	Liaoning	R	6	59.3	158	114	44	Chinese

334	Pan GF	2020	Fujian	P	7	54.1	70	41	29	Chinese
335	Chen H	2020	Jiangsu	R	6	NA	221	163	58	Chinese
336	Chen TY	2020	Guangdong	P	6	65.2	132	81	51	Chinese
337	Jiao HD	2020	Henan	P	7	54.3	150	81	69	Chinese
338	Hou Y	2020	Jiangsu	P	6	66	90	49	41	Chinese
339	Liao H	2020	Shanghai	P	6	46	128	99	29	Chinese
340	Yuan GF	2020	Jiangsu	P	7	57.4	68	41	27	Chinese
341	Liu Y	2020	Henan	P	6	62.9	86	31	55	Chinese
342	Chen ZQ	2020	Henan	P	5	50.4	72	41	31	Chinese
343	Zhao Y	2020	Liaoning	R	7	63	194	142	52	Chinese
344	Guo YH	2020	Shaanxi	P	7	54.6	46	27	19	Chinese
345	Yuan Q	2020	Gansu	P	6	59.9	90	51	39	Chinese
346	Liu XF	2020	Hebei	P	7	NA	79	NA	NA	Chinese
347	Liu J	2020	Qinghai	P	6	53	30	19	11	Chinese
348	Wang DJ	2020	Jiangsu	P	7	58.2	66	37	29	Chinese
349	Zhang L	2020	Shaanxi	R	7	57.9	720	543	177	Chinese
350	Cui YF	2020	Shanxi	R	6	59.1	158	91	67	Chinese
351	Wu MH	2020	Jilin	R	6	60.5	842	629	213	Chinese
352	Yu Y	2020	Hebei	R	7	NA	212	152	60	Chinese
353	Wang QF	2020	Jiangxi	P	7	60.1	80	43	37	Chinese
354	Li Y	2020	Henan	P	6	59.8	92	59	33	Chinese
355	Hu B	2020	Henan	P	6	69.1	124	68	56	Chinese
356	Xia HY	2020	Fujian	P	6	51.4	68	39	29	Chinese
357	Yi QH	2020	Xinjiang	R	6	NA	76	53	23	Chinese
358	Chen Y	2021	Shaanxi	P	6	56.8	150	90	60	Chinese
359	Li FJ	2021	Shandong	R	5	NA	364	278	86	Chinese
360	Ding PA	2021	Hebei	P	6	NA	1347	NA	NA	Chinese
361	Ai Y	2021	Fujian	R	6	59.6	65	40	25	Chinese
362	Zheng WJ	2021	Fujian	P	6	60.1	80	37	43	Chinese
363	Zhao LP	2021	Zhejiang	R	6	57.8	69	43	26	Chinese
364	Huang WC	2021	Fujian	R	7	64	126	110	16	Chinese
365	Gao GM	2021	Jiangxi	R	6	NA	1256	NA	NA	Chinese
366	Wang XD	2021	Qinghai	P	6	58.3	116	75	41	Chinese
367	Ding PA	2021	Hebei	R	6	NA	558	NA	NA	Chinese
368	Huang WC	2021	Fujian	P	6	56.9	110	59	51	Chinese
369	He HS	2021	Fujian	R	6	66.9	70	37	33	Chinese
370	Jia YX	2021	Henan	P	6	NA	102	79	23	Chinese
371	Lv J	2021	Shaanxi	R	6	56.8	105	54	51	Chinese
372	Fang Z	2021	Shandong	R	6	65.9	183	137	46	Chinese
373	Yuan Y	2021	Shanghai	P	6	NA	81	NA	NA	Chinese
374	Wei SH	2021	Fujian	R	6	57	25	19	6	Chinese
375	Cui H	2021	Beijing	R	7	NA	130	NA	NA	Chinese

376	Li YF	2021	Shaanxi	P	6	63.4	84	53	31	Chinese
377	Du GJ	2021	Shandong	R	7	NA	303	213	90	Chinese
378	Zhang J	2021	Jiangsu	P	6	67.8	487	378	109	Chinese
379	Xiao YP	2021	Hunan	R	6	55.4	2029	1332	697	Chinese
380	Kamili	2021	Xinjiang	P	6	60.2	166	123	43	Chinese
381	Han SF	2021	Liaoning	R	6	63	195	138	57	Chinese
382	Li AQ	2021	Anhui	P	6	NA	900	682	218	Chinese
383	Zhang Y	2021	Beijing	P	6	56.4	116	61	55	Chinese
384	Ji HY	2021	Jiangsu	P	7	55.4	80	46	34	Chinese
385	Zhang QL	2021	Hebei	R	6	60.3	398	262	136	Chinese
386	Zhang GH	2021	Sichuan	R	6	50.9	208	120	88	Chinese
387	Ding PA	2021	Hebei	P	6	NA	277	NA	NA	Chinese
388	Ma J	2021	Anhui	R	7	62.5	525	387	138	Chinese
389	Gao B	2021	Liaoning	P	5	62.1	174	116	58	Chinese
390	Fu YN	2021	Liaoning	R	6	NA	197	103	94	Chinese
391	Meng N	2021	Hebei	R	6	NA	72	NA	NA	Chinese
392	Li C	2021	Henan	R	6	NA	34	24	10	Chinese
393	Huang WB	2021	Hubei	P	6	50.8	96	68	28	Chinese
394	Zhang YF	2021	Beijing	R	6	57.2	166	102	64	Chinese
395	Tian Y	2021	Hebei	P	6	NA	132	119	13	Chinese
396	Wang TB	2021	Beijing	R	6	55	490	358	132	Chinese
397	Chen XM	2021	Ningxia	P	6	NA	179	112	67	Chinese
398	Xu L	2021	Sichuan	P	6	61	86	48	38	Chinese
399	Li MZ	2021	Tianjin	R	6	NA	68	25	43	Chinese
400	Hu XF	2021	Henan	R	7	NA	86	38	48	Chinese
401	Xia L	2021	Anhui	R	6	NA	86	90	26	Chinese
402	Ma YW	2021	Gansu	R	6	NA	1639	NA	NA	Chinese
403	Li HZ	2021	Henan	P	6	NA	255	218	37	Chinese
404	Ni T	2021	Guangxi	P	7	62	69	61	8	Chinese
405	Wang RJ	2021	Shandong	P	8	NA	120	86	34	Chinese
406	Yan Q	2021	Guangdong	R	7	NA	131	100	31	Chinese
407	Huang ZP	2021	Gansu	P	6	NA	665	509	156	Chinese
408	Cui WH	2021	Shandong	P	7	NA	719	514	205	Chinese
409	Shu HG	2021	Jiangxi	P	7	61.8	44	37	7	Chinese
410	Zhao Y	2021	Shandong	P	7	61.7	223	195	28	Chinese
411	Wang MY	2021	Anhui	R	7	NA	40	30	10	Chinese
412	Zhang HY	2021	Anhui	R	6	62.2	24	20	4	Chinese
413	Ma S	2021	Liaoning	R	7	NA	2526	NA	NA	Chinese
414	Li GG	2021	Qinghai	R	7	NA	156	NA	NA	Chinese
415	Yang SN	2021	Henan	P	7	NA	73	51	22	Chinese
416	Zhu H	2021	Jiangsu	P	7	NA	108	69	39	Chinese
417	Zheng H	2021	Anhui	P	6	60	80	53	27	Chinese

418	Zhang MK	2021	Shandong	R	6	61.9	80	60	20	Chinese
419	Cheng J	2021	Hubei	P	6	48.3	70	43	27	Chinese
420	Chen W	2021	Jiangsu	R	6	42.7	120	74	46	Chinese
421	Qi S	2021	Sichuan	P	6	51.6	70	59	11	Chinese
422	Ji SW	2021	Shanxi	P	5	62.3	80	47	33	Chinese
423	Kong XC	2021	Shanghai	R	6	NA	147	NA	NA	Chinese
424	Lin RG	2021	Guangdong	P	5	NA	48	NA	NA	Chinese
425	Wang HM	2021	Shandong	P	6	61.4	104	63	41	Chinese
426	Han S	2021	Hubei	P	5	47.5	82	43	39	Chinese
427	Wang HW	2021	Guangdong	P	6	56.5	60	33	27	Chinese
428	Zhang J	2021	Henan	R	6	58.2	88	58	30	Chinese
429	Wang Z	2021	Anhui	P	6	64	40	33	7	Chinese
430	Chen YJ	2021	Xinjiang	R	5	66.2	36	26	10	Chinese
431	Fu YJ	2021	Shanghai	R	6	64.2	535	389	146	Chinese
432	Xie YY	2021	Inner Mongolia	P	6	72.2	404	255	149	Chinese
433	Meng M	2021	Shandong&Zhejiang	R	5	58.1	76	46	30	Chinese
434	Chen ZL	2021	Henan	P	6	52.5	84	57	27	Chinese
435	Ding DQ	2021	Anhui	P	7	63.7	72	38	34	Chinese
436	Han YH	2021	Shandong	P	6	61.3	80	48	32	Chinese
437	Chao LM	2021	Inner Mongolia	P	6	45.7	78	44	34	Chinese
438	Guo YM	2021	Shaanxi	P	7	52.8	183	114	69	Chinese
439	Yu DZ	2021	Hebei	P	6	65.4	83	54	29	Chinese
440	Xiao ZJ	2021	Inner Mongolia	R	6	NA	80	56	24	Chinese
441	Yang WB	2021	Hunan	P	6	NA	96	58	38	Chinese
442	Zhong XS	2021	Guangdong	P	6	58.6	82	46	36	Chinese
443	Li LP	2021	Guangdong	P	7	49.6	97	62	35	Chinese
444	Du CR	2021	Anhui	P	6	59.1	65	36	29	Chinese
445	Miao XW	2021	Shaanxi	R	8	NA	636	504	132	Chinese
446	Hu GP	2021	Qinghai	P	8	57	600	454	146	Chinese
447	Liu QQ	2021	Xinjiang	P	7	NA	160	NA	NA	Chinese
448	Zhang ZZ	2021	Hebei	P	8	58.2	70	54	16	Chinese
449	Feng S	2021	Anhui	R	6	NA	188	104	84	Chinese
450	Tang Q	2022	Hunan	R	7	51.6	385	215	170	Chinese
451	Wang CY	2016	Fujian	R	5	NA	65	38	27	Chinese
452	Su HY	2016	Jiangsu	R	6	NA	717	516	201	Chinese
453	Yang WY	2016	Shanxi	R	7	42.4	84	60	24	Chinese
454	Yu WW	2016	Gansu	R	6	NA	47	38	9	Chinese
455	Liu YL	2016	Beijing	R	6	NA	321	168	153	Chinese
456	Zhang Y;	2016	Shanghai	R	5	NA	128	71	57	Chinese
457	Li XL	2016	Shaanxi	P	6	58.1	116	76	40	Chinese
458	Chen YW	2016	Anhui	R	5	51.1	90	56	34	Chinese
459	Bai JW	2016	Henan	R	7	NA	288	175	113	Chinese

460	Wang Y;	2016	Sichuan	R	6	63.3	78	42	36	Chinese
461	Zhang H	2016	Henan	R	7	NA	94	50	44	Chinese
462	Wang T;	2016	Henan	R	6	NA	60	46	14	Chinese
463	Li ZY	2016	Chongqing	R	6	NA	678	441	237	Chinese
464	Cao XL	2016	Beijing	R	7	NA	63	51	12	Chinese
465	Yang RL	2016	Guangxi	R	6	NA	130	81	49	Chinese
466	NA	2016	Guangdong&Xinjiang	R	6	NA	45	NA	NA	Chinese
467	Song ZY	2016	Jilin	R	7	NA	788	593	195	Chinese
468	Cheng YL	2016	Liaoning	R	7	57	610	426	184	Chinese
469	Li LS	2016	Gansu	P	7	NA	413	299	114	Chinese
470	Jin X;	2016	Shaanxi	R	6	54.5	280	181	99	Chinese
471	Yu WW	2016	Jiangsu	P	6	NA	64	43	21	Chinese
472	You XL	2016	Jiangsu	R	7	NA	126	87	39	Chinese
473	Zhuo CY	2016	heilongjiang	R	7	58.2	395	303	92	Chinese
474	Guo P;	2016	Hubei	P	5	NA	76	53	23	Chinese
475	Li C;	2016	Zhejiang	P	7	NA	120	68	52	Chinese
476	Yang J;	2016	Gansu	R	6	NA	287	227	60	Chinese
477	Su ZR	2016	Anhui	R	6	NA	61	42	19	Chinese
478	Zhao TP	2016	Gansu	P	7	NA	42	26	16	Chinese
479	Li JW	2016	Henan	P	5	NA	68	41	27	Chinese
480	Xu J;	2016	Hubei	R	5	56	86	50	36	Chinese
481	Guo YN	2016	Tianjin	R	6	NA	64	50	14	Chinese
482	Xu YW	2016	Fujian	P	6	NA	62	31	31	Chinese
483	Zhao YY	2016	Liaoning	R	8	58.3	505	369	136	Chinese
484	Xie WL	2016	Jiangxi	R	6	NA	18	13	5	Chinese
485	Zhang Y;	2016	Beijing	R	6	NA	144	NA	NA	Chinese
486	Ma WQ	2016	Hebei	P	7	NA	130	104	26	Chinese
487	Tan M	2016	Hebei	P	8	57.2	140	108	32	Chinese
488	Xu WZ	2016	Hebei	P	7	NA	80	50	30	Chinese
489	Qiu H	2016	Guangxi	R	7	56.8	257	178	79	Chinese
490	Zhang YL	2016	Jiangsu	R	6	NA	131	69	62	Chinese
491	Zou ZY	2016	Beijing	R	7	NA	245	160	85	Chinese
492	Yang AG	2016	Fujian	R	6	NA	39	NA	NA	Chinese
493	Hu JJ	2016	Sichuan	R	7	NA	73	42	31	Chinese
494	Shen XY	2016	Liaoning	P	6	NA	108	61	47	Chinese
495	Geng W	2016	Jiangsu	R	7	NA	46	36	10	Chinese
496	Wang HY	2016	Hubei	R	6	NA	80	51	29	Chinese
497	Shang YC	2016	Shaanxi	P	6	NA	90	60	30	Chinese
498	Zhong JF	2016	Shanxi	P	6	NA	76	49	27	Chinese
499	Zhu CC	2016	Shanghai	R	6	62.2	3121	2125	996	Chinese
500	Li DY	2016	Guangdong	R	6	58.7	119	73	46	Chinese
501	Xia QY	2016	Jiangsu	P	5	54.5	48	29	19	Chinese

502	Xuan ZQ	2017	Jiangsu	R	6	NA	105	63	42	Chinese
503	Zhang MY	2017	Liaoning	R	7	60.5	150	85	65	Chinese
504	Yang T;	2017	Fujian	R	6	NA	55	26	29	Chinese
505	Ge XS	2017	Jiangsu	R	7	NA	76	63	13	Chinese
506	Kong XJ	2017	Shanghai	R	8	60.2	273	184	89	Chinese
507	Qi C	2017	Shandong	R	7	56	61	43	18	Chinese
508	Xu HL	2017	Hubei	P	6	47.2	60	38	22	Chinese
509	Zhou HF	2017	Zhejiang	R	6	NA	436	311	125	Chinese
510	Mi HN	2017	Gansu	R	7	NA	117	82	35	Chinese
511	He X	2017	Jiangsu	R	8	NA	72	44	28	Chinese
512	Xie LH	2017	Guangdong	P	6	NA	62	38	24	Chinese
513	Han LL	2017	Henan	P	5	44.5	168	120	48	Chinese
514	Ba N;	2017	Henan	P	6	52.8	106	59	47	Chinese
515	Zhu XQ	2017	Jiangsu	R	7	NA	98	82	16	Chinese
516	Dou HM	2017	Jiangsu	R	5	57	160	71	89	Chinese
517	Lu LH	2017	Jiangsu	R	6	NA	60	36	24	Chinese
518	Zhong LX	2017	Fujian	R	5	71.2	68	35	33	Chinese
519	Jiang XM	2017	Zhejiang	P	6	NA	110	88	22	Chinese
520	Zhou JJ	2017	Zhejiang	P	7	NA	426	282	144	Chinese
521	Jiang WH	2017	Jiangsu	P	7	NA	44	27	17	Chinese
522	Liao HH	2017	Hunan	P	6	58.1	64	43	21	Chinese
523	Zheng YT	2017	Henan	P	6	NA	72	40	32	Chinese
524	Li X;	2017	Beijing	P	7	NA	108	57	49	Chinese
525	Wei QW	2017	Hubei	R	6	NA	136	75	61	Chinese
526	Yu M;	2017	Zhejiang	R	7	NA	338	NA	NA	Chinese
527	Cheng YS	2017	Anhui	R	7	NA	107	86	21	Chinese
528	Yu WW	2017	Gansu	R	7	75.8	33	NA	NA	Chinese
529	Wang XJ	2017	Fujian	P	6	NA	85	47	38	Chinese
530	Wang BY	2017	Jiangsu	R	7	NA	109	82	27	Chinese
531	Su L;	2017	Gansu	R	6	NA	95	NA	NA	Chinese
532	Zhou T;	2017	Shandong	R	6	NA	114	94	20	Chinese
533	Niu ST	2017	Henan	R	6	52.7	58	31	27	Chinese
534	Zhan RY	2017	Sichuan	P	5	NA	60	35	25	Chinese
535	He Y	2017	Henan	P	6	NA	96	53	43	Chinese
536	Zhang X;	2017	heilongjiang	P	8	NA	255	181	74	Chinese
537	Hu SB	2017	Beijing	R	8	NA	87	66	21	Chinese
538	Zhang MJ	2017	Henan	P	6	59.5	42	NA	NA	Chinese
539	He ZR	2017	Hubei	R	5	NA	50	27	23	Chinese
540	Ren H	2017	Beijing	R	7	62.1	95	80	15	Chinese
541	Song JW	2017	heilongjiang	R	6	NA	115	NA	NA	Chinese
542	Zhang SB	2017	Shaanxi	P	7	NA	120	54	66	Chinese
543	She XC	2017	Guangdong	P	6	NA	36	21	15	Chinese

544	Dong QF	2017	Zhejiang	R	6	NA	60	33	27	Chinese
545	Zhang F;	2017	Sichuan	P	6	NA	116	69	47	Chinese
546	Hu JW	2017	Henan	R	6	60.5	298	226	72	Chinese
547	Peng WX	2017	Anhui	R	7	61.4	290	178	52	Chinese
548	Song Z;	2017	Henan	R	7	NA	78	62	16	Chinese
549	Chen LC	2017	Fujian	R	7	NA	1629	NA	NA	Chinese
550	Wang JB	2017	Fujian	R	7	NA	1568	NA	NA	Chinese
551	Chen ZP	2017	Fujian	R	7	NA	86	49	37	Chinese
552	Yang AF	2017	Henan	P	6	NA	80	47	33	Chinese
553	Gao XY	2017	Fujian	P	6	NA	123	74	49	Chinese
554	Zhang X;	2017	heilongjiang	P	6	NA	255	181	74	Chinese
555	Li ZQ	2017	Shandong	R	8	56.2	275	202	73	Chinese
556	Hu X;	2017	Sichuan	R	7	58.3	143	98	45	Chinese
557	Sun XZ	2017	Tianjin	R	5	63.1	800	472	328	Chinese
558	Zhou B	2017	Henan	P	6	NA	114	69	45	Chinese
559	Shi XJ	2017	Jiangsu	R	6	NA	46	NA	NA	Chinese
560	Shen GJ	2017	Zhejiang	R	6	60	73	52	21	Chinese
561	Li L	2017	Liaoning	P	7	NA	160	86	74	Chinese
562	Huang QX	2017	Shanxi	R	6	NA	142	NA	NA	Chinese
563	Li SW	2017	Jiangsu	R	7	NA	77	NA	NA	Chinese
564	Lin CZ	2017	Fujian	R	7	NA	94	69	25	Chinese
565	Shen Y	2017	Fujian	R	8	NA	259	NA	NA	Chinese
566	Liang P;	2017	Liaoning	P	8	55	50	31	19	Chinese
567	Du XC	2017	Henan	R	7	NA	64	41	23	Chinese
568	Yuan QQ	2017	Anhui	R	7	60	276	213	63	Chinese
569	Wang FB	2017	Jiangsu	P	6	NA	100	68	32	Chinese
570	Jia TY	2017	Henan	R	7	NA	100	58	42	Chinese
571	Wang HR	2017	Shandong	R	6	NA	60	NA	NA	Chinese
572	Gu T	2017	Zhejiang	R	7	NA	60	NA	NA	Chinese
573	Li XY	2017	Zhejiang	R	7	62.5	120	92	28	Chinese
574	Wang F	2017	Henan	R	6	NA	94	57	37	Chinese
575	Peng Q;	2017	Fujian	P	6	NA	48	33	15	Chinese
576	Yao Q;	2018	Jiangsu	R	6	73.8	146	79	67	Chinese
577	Tang J;	2018	Hunan	R	5	55	115	66	49	Chinese
578	Li M	2018	Jiangxi	P	7	NA	186	130	56	Chinese
579	Yu WB	2018	Fujian	R	7	59.6	54	46	8	Chinese
580	Liu Y	2018	Jiangxi	R	7	60	247	169	51	Chinese
581	Cao YX	2018	Jiangsu	R	8	NA	163	116	47	Chinese
582	Hao LJ	2018	Shandong	P	7	63.5	38	21	17	Chinese
583	Fu L	2018	Henan	R	7	NA	62	41	21	Chinese
584	Zhao WF	2018	Henan	P	7	NA	96	55	41	Chinese
585	Zhou ZW	2018	Anhui	R	6	61.6	117	84	33	Chinese

586	Zhang HL	2018	Zhejiang	R	6	NA	940	667	273	Chinese
587	Yao ZD	2018	Beijing	R	6	NA	188	141	47	Chinese
588	Chen ZX	2018	Jiangxi	P	6	NA	75	57	18	Chinese
589	Zhang KC	2018	Beijing	R	8	NA	140	NA	NA	Chinese
590	Li M;	2018	Qinghai	P	7	NA	92	53	39	Chinese
591	Guo MK	2018	Anhui	P	6	NA	62	38	24	Chinese
592	Jia JR	2018	Hubei	R	8	58.6	90	66	24	Chinese
593	Yu H	2018	Jiangsu	R	6	NA	42	29	13	Chinese
594	Lin DJ	2018	Fujian	R	5	58.3	183	121	62	Chinese
595	Zheng ZG	2018	Fujian	P	6	NA	78	45	33	Chinese
596	Zhao YH	2018	Gansu	R	5	NA	52	27	25	Chinese
597	Ye LL	2018	Anhui	R	8	NA	64	43	21	Chinese
598	Shang DF	2018	Henan	P	8	NA	96	66	30	Chinese
599	Zhang XJ	2018	Jiangsu	R	6	NA	80	33	47	Chinese
600	Xie YR	2018	Hubei	R	7	NA	100	56	44	Chinese
601	Xiao R	2018	Fujian	R	7	NA	74	NA	NA	Chinese
602	Shang L;	2018	Shaanxi	R	5	NA	469	360	109	Chinese
603	Zhu H;	2018	Anhui	R	6	NA	1700	NA	NA	Chinese
604	Cheng HF	2018	Guangxi	R	8	58.3	144	101	43	Chinese
605	Bai BL	2018	Zhejiang	P	7	63.8	182	138	44	Chinese
606	Zhao YB	2018	Liaoning	P	6	NA	60	43	17	Chinese
607	Wang HH	2018	Liaoning	P	8	51	50	32	18	Chinese
608	Song CJ	2018	Jiangsu	R	5	62.4	102	NA	NA	Chinese
609	Zhou H;	2018	Jiangsu	R	6	NA	147	107	40	Chinese
610	Chang N	2018	Henan	R	7	55.5	180	109	71	Chinese
611	Liu CC	2018	Anhui	R	5	58.6	42	32	10	Chinese
612	Wang Y	2018	Hebei	R	7	NA	106	68	38	Chinese
613	Wei ZJ	2018	Anhui	R	6	NA	198	NA	NA	Chinese
614	Ji ZH	2018	Beijing	P	7	NA	110	52	58	Chinese
615	Wang SC	2018	Shanghai	P	7	62.2	4311	2942	1369	Chinese
616	Ji W	2018	Beijing	P	7	NA	26	NA	NA	Chinese
617	Li RD	2018	Jilin	P	6	NA	181	145	36	Chinese
618	Nie L;	2018	Fujian	P	7	NA	60	37	23	Chinese
619	Chen LC	2018	Fujian	R	7	58.9	1801	1292	509	Chinese
620	Qu AY	2018	Liaoning	P	6	68.1	44	31	13	Chinese
621	Di HF	2018	Hebei	R	6	NA	90	52	38	Chinese
622	Sun SQ	2018	HeiLongJiang	P	8	NA	80	45	35	Chinese
623	Wang HB	2018	Chongqing	P	6	NA	60	33	27	Chinese
624	Zhang LX	2018	Anhui	R	8	NA	132	106	26	Chinese
625	Wang L	2018	Liaoning	R	8	NA	73	51	22	Chinese
626	Gao P;	2018	Jiangxi	R	7	NA	80	58	22	Chinese
627	Wang C	2018	Shandong	R	7	NA	95	65	30	Chinese

628	Song YJ	2018	Hubei	P	7	NA	107	69	38	Chinese
629	Zhang YX	2018	Henan	R	6	NA	113	66	47	Chinese
630	Wang HX	2018	Shaanxi	P	6	NA	77	57	20	Chinese
631	Li JL	2018	Shanxi	R	6	NA	60	36	24	Chinese
632	Wang Y	2018	Tianjin	R	6	NA	142	101	41	Chinese
633	Wang MK	2018	Guizhou	R	7	55.9	85	57	28	Chinese
634	Yang L;	2018	Shaanxi	R	6	NA	120	71	49	Chinese
635	Liu F	2018	Jilin	P	6	NA	98	53	45	Chinese
636	Zhang HQ	2018	Shaanxi	R	8	NA	128	NA	NA	Chinese
637	Cai FB	2018	Fujian	R	7	NA	128	96	32	Chinese
638	Yang JF	2018	Henan	P	6	NA	56	39	17	Chinese
639	Zhu SY	2018	Zhejiang	P	6	NA	60	23	37	Chinese
640	Tian H	2018	Liaoning	R	6	NA	96	NA	NA	Chinese
641	Yao GY	2018	Zhejiang	P	6	NA	86	52	34	Chinese
642	Bi LQ	2018	Jiangsu	R	6	NA	70	39	31	Chinese
643	Yu WB	2018	Fujian	P	7	59.6	54	46	8	Chinese
644	Liu JS	2018	Shaanxi	R	6	57.5	79	68	11	Chinese
645	Wei GB	2018	Shaanxi	R	5	NA	98	NA	NA	Chinese
646	You YZ	2018	Guangdong	P	7	NA	153	NA	NA	Chinese
647	Liao L;	2018	Hunan	R	6	NA	94	61	33	Chinese
648	He J;	2018	Sichuan	R	6	NA	100	51	49	Chinese
649	Yin TS	2018	Sichuan	R	6	NA	70	38	32	Chinese
650	Qu YL	2019	Xinjiang	R	5	43	29	0	29	Chinese
651	Sai FD	2019	Xinjiang	R	5	59.8	42	28	14	Chinese
652	Deng WF	2019	Guangdong	P	6	NA	50	29	21	Chinese
653	Wei XB	2019	Guangxi	R	5	NA	102	63	39	Chinese
654	Li ZG	2019	Anhui	R	6	NA	39	24	15	Chinese
655	Guo YT	2019	Shanxi	R	7	NA	183	115	68	Chinese
656	Zhou RJ	2019	Shanghai	R	6	61	278	207	74	Chinese
657	Nie HJ	2019	Henan	R	6	NA	89	52	37	Chinese
658	Liu LJ	2019	Hainan	R	6	NA	312	217	95	Chinese
659	Xu ZP	2019	Jiangsu	P	6	NA	404	NA	NA	Chinese
660	Yang Y;	2019	Beijing	P	6	NA	3241	2363	878	Chinese
661	Du J;	2019	Hubei	R	5	NA	104	62	42	Chinese
662	Luo R;	2019	Liaoning	R	5	56.2	50	38	12	Chinese
663	Lin JX	2019	Fujian	R	6	NA	4250	NA	NA	Chinese
664	Zhi ZY	2019	Henan	R	7	NA	268	NA	NA	Chinese

P, prospective study; R, retrospective study; NA, not available

Table S5 Pooled survival rates (%) and 95% CIs of gastric cancer in four time periods

**Survival**	**2000-2005**		**2006-2010**		**2011-2015**		**2016-2022**	
	N	SR (95% CI)	N	SR (95% CI)	N	SR (95% CI)	N	SR (95% CI)
1-year	33	76.8(71.5-82.1)	67	77.9(75.0-80.9)	139	75.5(73.2-77.7)	130	73.7(71.1-76.3)
2-year	12	48.8(34.5-63.1)	20	54.5(43.8-65.3)	73	57.2(50.3-64.1)	67	52.1(44.9-59.4)
3-year	36	49.3(41.5-57.1)	67	49.9(43.7-56.2)	143	54.3(49.1-59.5)	92	56.0(50.9-61.0)
5-year	51	44.5(38.2-50.8)	83	37.7(33.5-41.9)	115	45.9(41.5-50.3)	46	53.1(44.8-61.4)

N, numbers of included studies; SR, survival rate; CI, confidence interval.

[Supplementary figures]

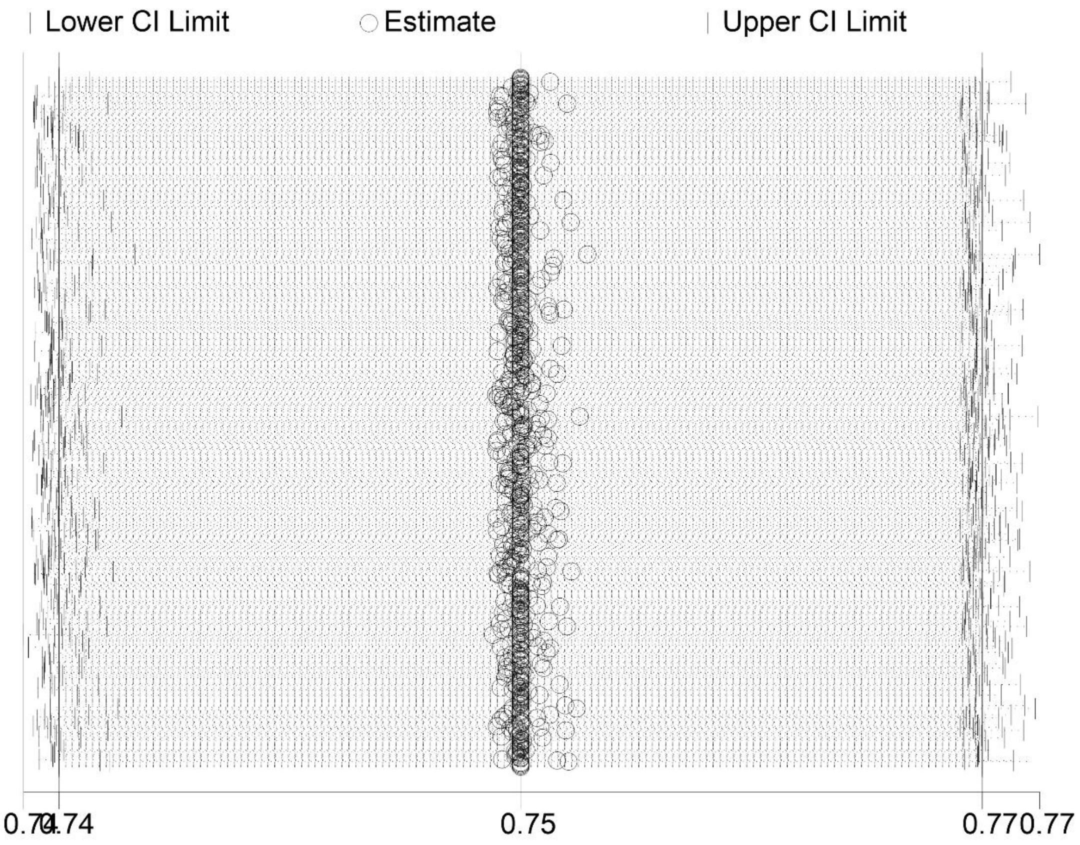

Figure S1 Sensitivity analysis for meta-analysis of one-year survival

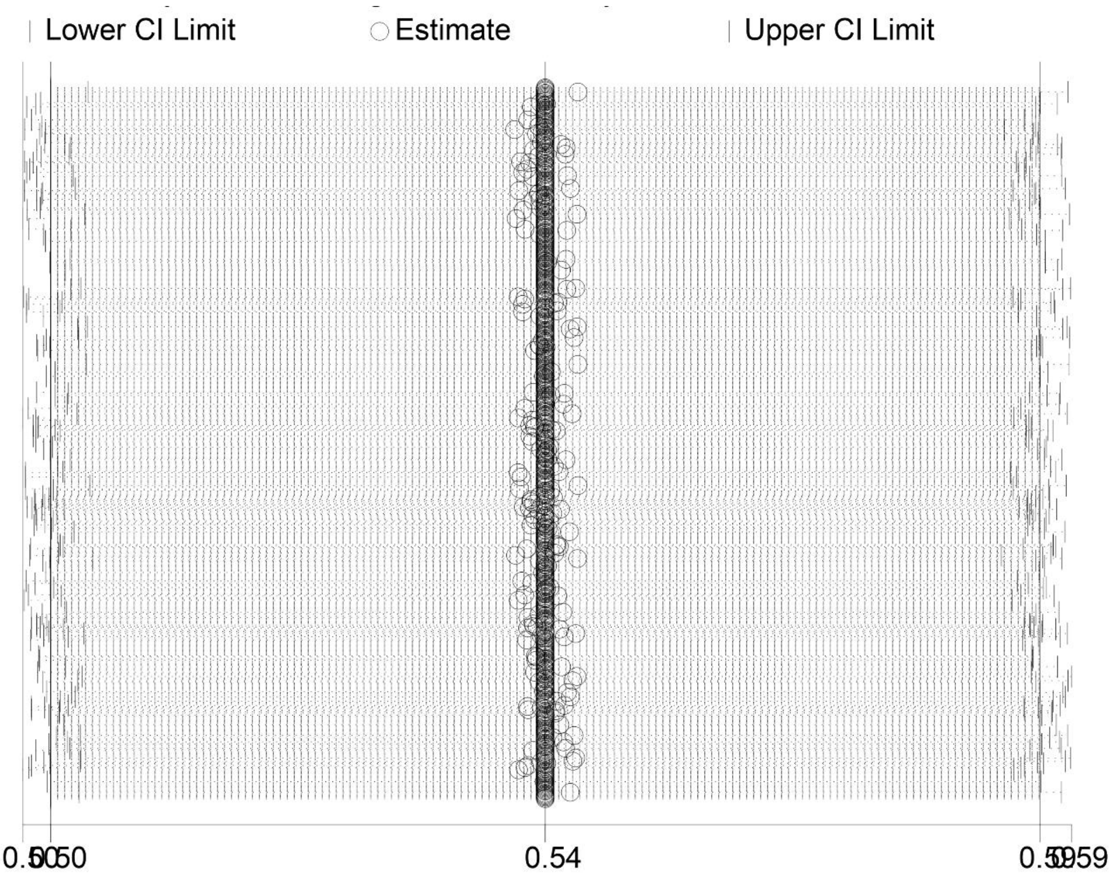

Figure S2 Sensitivity analysis for meta-analysis of two-year survival

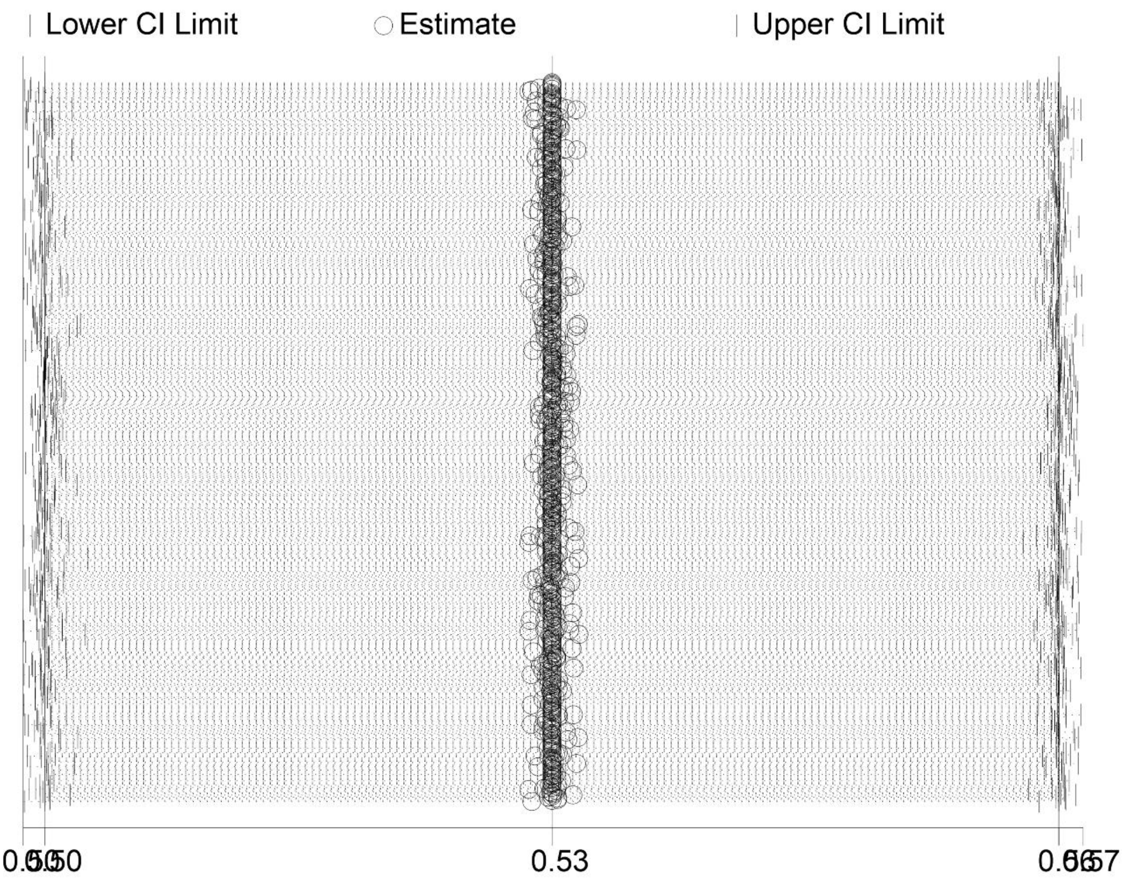

Figure S3 Sensitivity analysis for meta-analysis of three-year survival

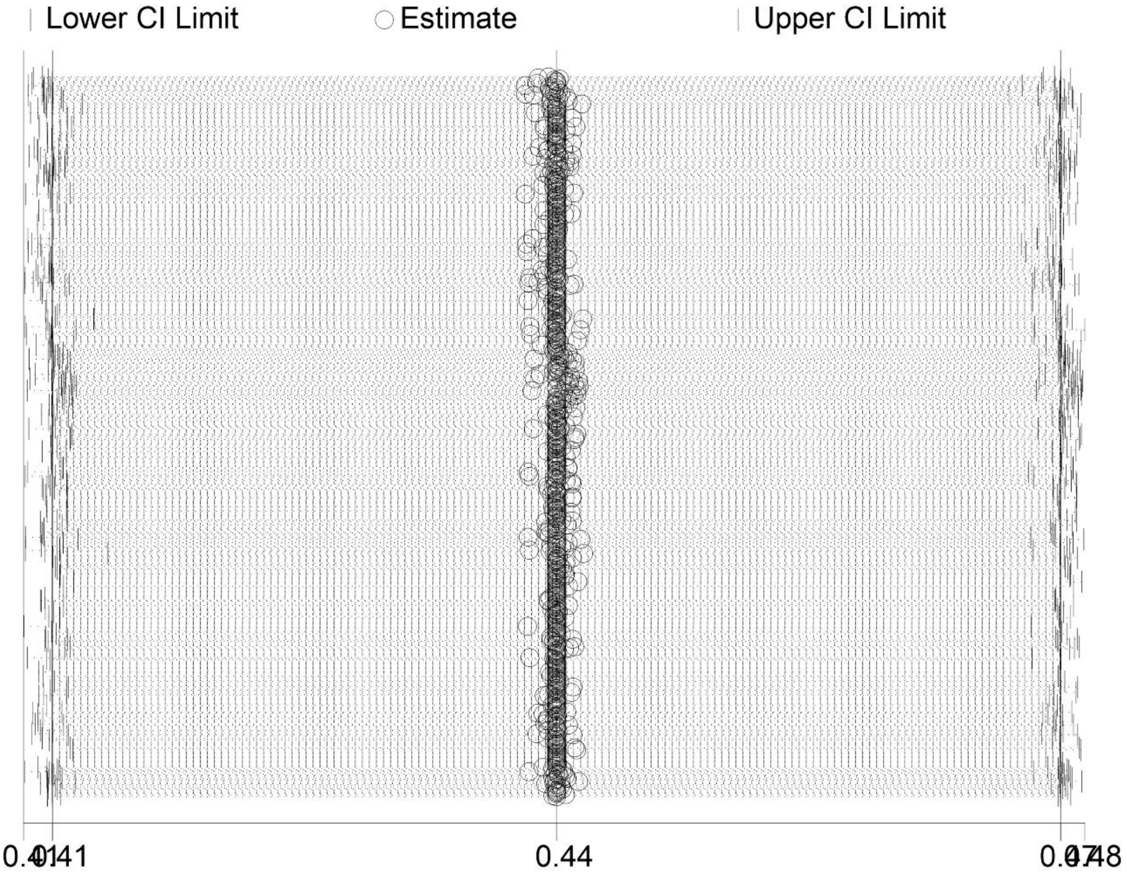

Figure S4 Sensitivity analysis for meta-analysis of five-year survival

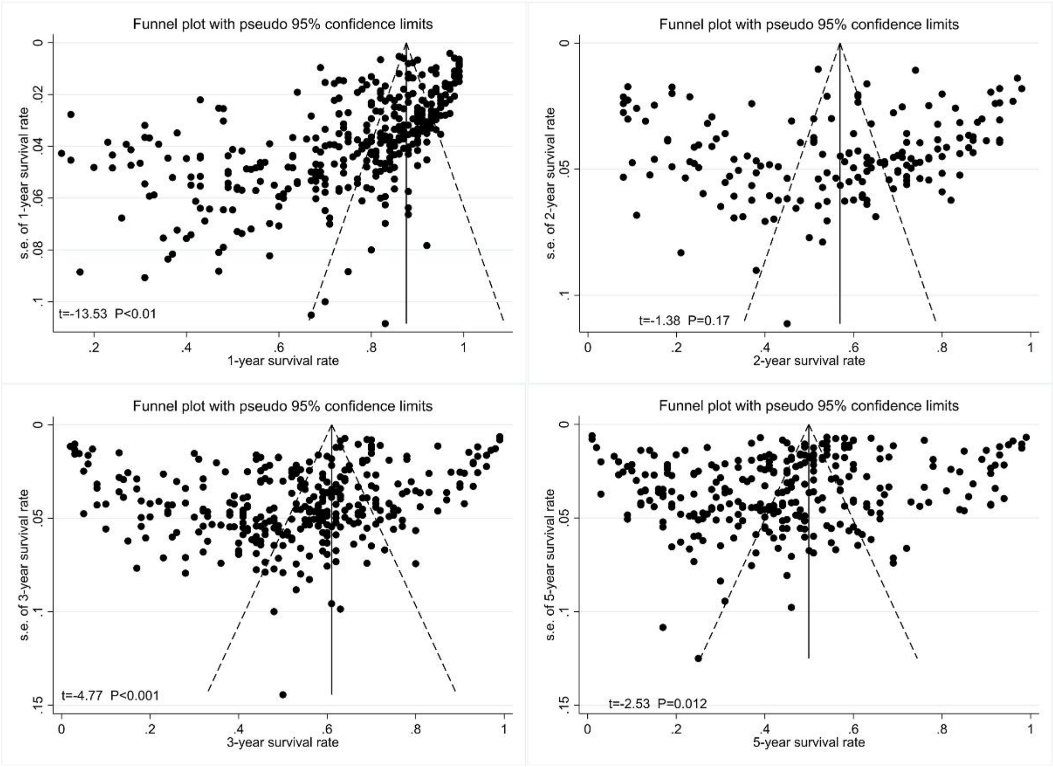

Figure S5 Funnel plots of publication bias analyses
